# A landscape‐scale assessment of the relationship between grassland functioning, community diversity, and functional traits

**DOI:** 10.1002/ece3.6650

**Published:** 2020-08-16

**Authors:** Hanneke van 't Veen, Loïc Chalmandrier, Nadine Sandau, Michael P. Nobis, Patrice Descombes, Achilleas Psomas, Yann Hautier, Loïc Pellissier

**Affiliations:** ^1^ Earth System Science Department of Geography University of Zurich (UZH) Zürich Switzerland; ^2^ School of Biological Sciences University of Canterbury Christchurch New Zealand; ^3^ LANAT Amt für Landwirtschaft und Natur Münsingen Switzerland; ^4^ Swiss Federal Research Institute WSL Birmensdorf Switzerland; ^5^ Ecology and Biodiversity Group Department of Biology Utrecht University Utrecht The Netherlands; ^6^ Landscape Ecology Institute of Terrestrial Ecosystems ETH Zürich Zürich Switzerland

**Keywords:** BEF, biodiversity, drought, ecosystem functioning, ecosystem services, grasslands, plant, productivity, trait composition

## Abstract

Livestock farmers rely on a high and stable grassland productivity for fodder production to sustain their livelihoods. Future drought events related to climate change, however, threaten grassland functionality in many regions across the globe. The introduction of sustainable grassland management could buffer these negative effects. According to the biodiversity–productivity hypothesis, productivity positively associates with local biodiversity. The biodiversity–insurance hypothesis states that higher biodiversity enhances the temporal stability of productivity. To date, these hypotheses have mostly been tested through experimental studies under restricted environmental conditions, hereby neglecting climatic variations at a landscape‐scale. Here, we provide a landscape‐scale assessment of the contribution of species richness, functional composition, temperature, and precipitation on grassland productivity. We found that the variation in grassland productivity during the growing season was best explained by functional trait composition. The community mean of plant preference for nutrients explained 24.8% of the variation in productivity and the community mean of specific leaf area explained 18.6%, while species richness explained only 2.4%. Temperature and precipitation explained an additional 22.1% of the variation in productivity. Our results indicate that functional trait composition is an important predictor of landscape‐scale grassland productivity.

## Graphical abstract

We provide a landscape‐scale assessment on the relative effect of plant species richness and plant trait composition on the productivity and variance in grassland productivity under varying climatic conditions in Switzerland. We find a relatively strong effect of functional traits on grassland productivity, while species richness slightly influences productivity. The relationships between functional traits on grassland productivity found in our study indicate the importance of including functional trait variables to explain productivity on a landscape scale.

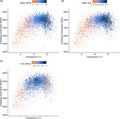

## 1 INTRODUCTION

Livestock farmers around the globe rely on high and stable grassland productivity for fodder production (FSO, 2015). To boost the productivity of their land, farmers frequently use fertilizers and seed mixes (FSO, 2015). A side effect of this approach is a loss of plant biodiversity and a transition toward productive species that rely on abundant resources to survive (Flynn et al., 2009; Gross & Mittelbach, 2017; Harpole & Tilman, 2007; Socher et al., 2012). This is concerning, because the loss of diversity could reduce grassland productivity and temporal stability of productivity in response to climatic perturbations, such as drought and flood events, which are becoming increasingly frequent worldwide (Hautier et al., 2015; Hector et al., 2010; IPCC, 2018; Isbell et al., 2013, 2015). Hence, intensive grassland management aimed at promoting productivity could potentially be counterproductive. It is vital to determine the relative contribution of biodiversity and climatic variability to grassland productivity and its temporal stability.

The positive effect of biodiversity on grassland productivity and temporal stability, as measured by the temporal mean of productivity divided by its temporal variability, can arise through multiple nonexclusive mechanisms. Niche complementarity can result in overyielding in a mixture when its biomass production exceeds that of the average monoculture of the species contained in the mixture (Schmid, Hector, Saha, & Loreau, 2008). Overyielding can contribute to increase the temporal stability of productivity by increasing the temporal mean of productivity (de Mazancourt et al., 2013). The portfolio effect (Doak et al., 1998) and insurance hypothesis (Yachi & Loreau, 1999) can contribute to reduce the temporal variability of productivity when decreases in the productivity of some species are compensated for by increases in other species. This asynchrony in species response to environmental fluctuations is more likely to occur when the species pool is larger and more diverse (Loreau, 2010). Additionally, temporal stability combines the effect of resistance and resilience (Isbell et al., 2015) and can arise because species‐rich communities are more likely to contain a mixture of productive species that recover well after a perturbation and stress‐tolerant species that resist well during a perturbation (Craven et al., 2018). Hence, biodiversity is expected to both boost productivity under average circumstances and stabilize productivity in response to environmental fluctuations (Hector et al., 1999; Isbell et al., 2015; Yachi & Loreau, 1999).

Recent experimental evidence suggests that in particular species, trait composition (i.e., the community‐weighted trait means and variances within a given area) promote grassland productivity (Gross et al., 2017; Tilman et al., 1997). The potential role of functional composition has already been demonstrated in several experimental field studies, where functional guilds have been manipulated (Gross et al., 2017; Marquard et al., 2009; Tilman et al., 1997). Although functional trait composition varies at any scale, contrasts between grasslands with very different trait compositions should be especially marked when studying entire landscapes, in particular since climate and management influence plant trait composition (Díaz & Cabido, 2001; Harpole & Tilman, 2007; Loreau et al., 2001). For example, grassland fertilization results in a dominance of fast‐growing species in grassland communities (Borer et al., 2014; Harpole & Tilman, 2007; Hautier, Niklaus, & Hector, 2009; Soons et al., 2017). Fast‐growing species have specific traits that distinguish them from stress‐tolerant species, such as a large specific leaf area and a preference for high nutrient levels (Mariotte, Vandenberghe, Kardol, Hagedorn, & Buttler, 2013). It is hypothesized that high investment in plant growth is at the expense of investment in stress tolerance (Willis, Thomas, & Lawton, 1999). At a landscape‐scale, the functional composition of grasslands could, therefore, be a profound predictor of both grassland productivity and temporal stability of grassland productivity (Fischer et al., 2016). Hence, to understand the influence of biodiversity on productivity at a landscape‐scale, it is important to include the functional trait composition of grassland communities (Loreau et al., 2001; Marquard et al., 2009).

Over the past decades, the majority of studies demonstrating diversity effects on productivity were conducted at a local scale, within a controlled setting of experiments under restricted environmental conditions (Hector et al., 1999; Reich et al., 2004; Roscher et al., 2004; Schittko, Hawa, & Wurst, 2014; Tilman et al., 1997). Experimental context can, however, significantly differ from situations in real landscapes (Sandau et al., 2017; Wardle, 2016). The relationship between biodiversity and productivity under natural conditions might be affected by a range of variables, including temperature and precipitation, which vary spatially, in topographically heterogeneous landscapes (Ciais et al., 2005; Mariotte et al., 2013). In addition, differences in regional species pools that arise from historical processes and management, such as grazing, mowing, the use of seed mixtures, and fertilizers, influence the species composition in grassland communities, which may affect productivity (Chalmandrier, Albouy, & Pellissier, 2017). Hence, in natural grasslands, changes in species composition in response to disturbances are nonrandom, while in experimental studies, species are randomly removed or combined from a species pool. To overcome this gap in the literature, researchers around the globe recently started to study the relative effect of biodiversity on productivity at a landscape‐scale (Grace et al., 2007, 2016; Loreau, Mouquet, & Gonzalez, 2003; Oehri, Schmid, Schaepman‐Strub, & Niklaus, 2017; Soliveres et al., 2016; Tylianakis et al., 2008; Winfree, Fox, Williams, Reilly, & Cariveau, 2015).

Previous studies found varying effects of biodiversity on ecosystem functioning in real‐world ecosystems (Grace et al., 2007; Grigulis et al., 2013; Loreau et al., 2003; Oehri et al., 2017; Soliveres et al., 2016; Tylianakis et al., 2008; Winfree et al., 2015). Both Oehri et al. (2017) and Tylianakis et al. (2008) showed a positive correlation between biodiversity and productivity at a landscape‐scale across a range of land use types, including forests, grasslands, and agricultural areas. Tylianakis et al. (2008), however, did not assess the relative effect of biodiversity in comparison to climatic variables, such as temperature and precipitation, while Oehri et al. (2017) did not consider a possible nonlinear effect of climate on productivity (Whittaker, 1960). Grace et al. (2007) did not find a significant effect of biodiversity on productivity at a landscape‐scale, therefore, hypothesizing that the overall influence of small‐scale biodiversity on productivity is weak. Grace et al. (2016) showed a positive relationship between diversity and productivity using small‐scale grassland plots around the world. Winfree et al. (2015) showed that not species richness but a few dominant species drive ecosystem functioning in natural ecosystems. A recent study compared the results of experimental studies with data collected in real‐world natural grassland communities (Jochum et al., 2019). Jochum et al. (2019) found that although variance in functional trait composition and species richness is higher in experimental grasslands, biodiversity‐ecosystem functioning relationships did not differ significantly between the two. The differences between the studies might be related to the varying spatial scales at which biodiversity–productivity relationships were assessed, which range from regional to national and global (Grace et al., 2007, 2016; Oehri et al., 2017; Winfree et al., 2015). Apart from Jochum et al. (2019), none of these studies found assessed the potential influence of functional trait composition on productivity and stability in grassland productivity. This is surprising, since there are indications that metrics of functional diversity are stronger predictors of ecosystem functioning than species richness in naturally assembled communities (van der Plas, 2019). Jochum et al. (2019) did not specifically assess biodiversity at a landscape‐scale, but focus on the differences between real‐world and experimental studies. Besides this, Grace et al. (2007) only assessed natural grasslands of which species compositions likely do not differ as fundamentally as those of grasslands used for livestock farming (Borer et al., 2014; Harpole & Tilman, 2007; Hautier et al., 2009; Soons et al., 2017). This indicates the need for a study that focuses on both the effect of species richness and functional trait composition on the productivity of grasslands over a topographically diverse landscape with strong biogeographical and management gradients. Today, novel remote sensing methods provide a major opportunity for the coupling of remotely sensed productivity and climate data with biodiversity measures to disentangle the effect of plant trait composition and climate variations on the productivity of grasslands at a landscape‐scale.

In this study, we evaluate the relative contribution of climate and biodiversity (species richness and functional trait composition) to productivity of grasslands across the topographically diverse landscape of Switzerland. We ask the following research questions: (a) Is productivity best explained by species richness, functional trait composition, or climatic variables at a landscape‐scale? and (b) Does biodiversity influence the stability of productivity under climatic variations? Our study differs from experimental studies in which biodiversity was artificially reduced, because experiments analyze the effect of random species removal on grassland productivity, while a study‐based data from grasslands at a landscape‐scale test the real‐life effect of existing grassland communities that differ in the amount of species and their functional trait distribution. We hypothesize that plant communities in agricultural areas dominated by productive species are more sensitive to climatic variation and thus less stable through time compared to communities composed of both productive and stress‐tolerant species (Schläpfer, Pfisterer, & Schmid, 2005; Wang, Yu, & Wang, 2007). We, furthermore, expect a trade‐off between the two (Willis et al., 1999). Overall, the results of this study could be integrated in management strategies in high‐intensity agricultural landscapes that are vulnerable to global changes.

## 2 MATERIALS AND METHODS

Our study focuses on Switzerland, a country with an area of ±41,000 km^2^ and strong gradients in elevation (190–4,500 m a.s.l.). Switzerland harbors intensive agricultural areas below the tree line (ca. 1,800 m a.s.l.) and 70.6% of the total agricultural area of Switzerland is covered by grasslands used for agriculture (FSO, 2015). To allow for a comparison between productivity, species richness, and the plant trait composition of grassland communities across Switzerland under varying climatic conditions, we combined databases on species occurrence, plant trait composition, grassland productivity, temperature, and precipitation. We processed all data in the software R (R Core Team, 2019) and ArcGIS 10.4.1 software (ESRI, Redlands, CA, USA, 2008).

### 2.1 Species occurrence data

**FIGURE 1:**
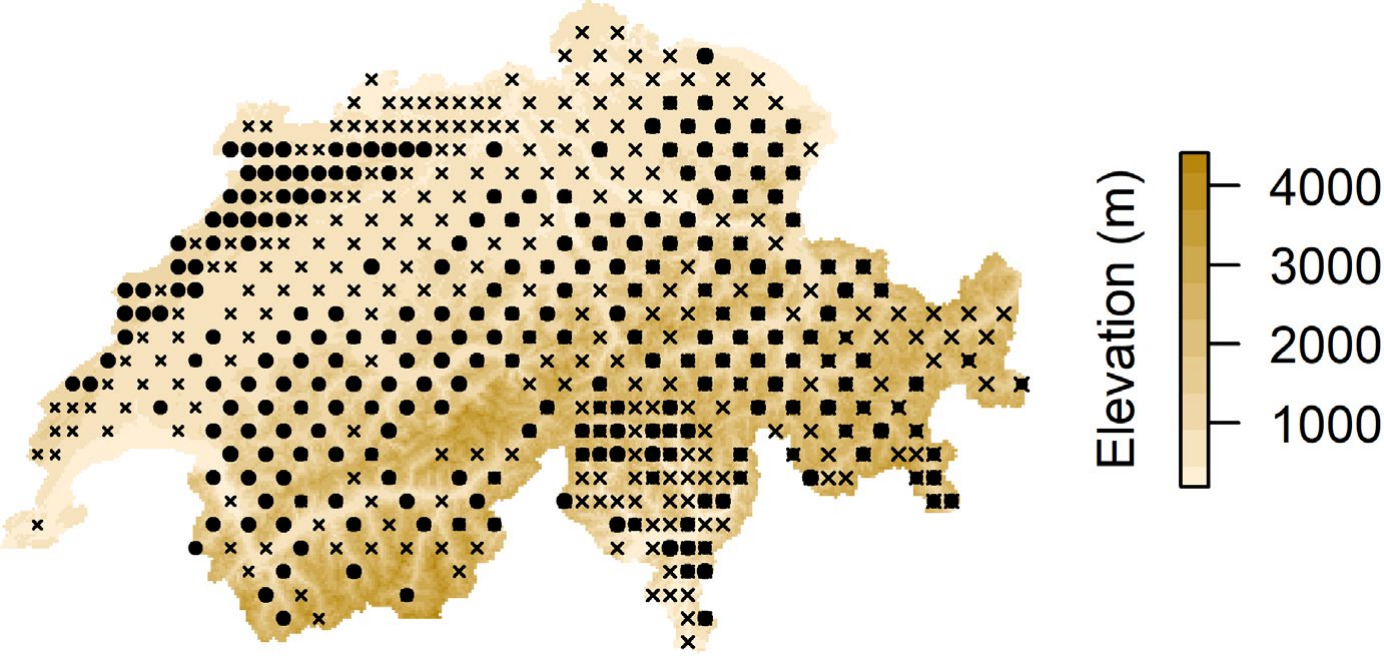
The 474 BDM plots distributed across Switzerland. The Biodiversity Monitoring Program of Switzerland collects species occurrence data of vascular plants in all 474 1‐by‐1 km BDM plots shown with black crosses (BDM, 2014). In this study, we solely assess grassland‐harboring BDM plots (277 plots shown with black dots)

We acquired species occurrence data from the Biodiversity Monitoring (BDM) Program (BDM, 2014). The BDM program (http://www.biodiversitymonitoring.ch) collects species occurrence data of vascular plants along transects of 2,500 m length within 474 1‐by‐1 km plots spread across Switzerland (Figure 1) (BDM, 2014). Each year, a subset of one fifth of all plots is surveyed. Since biodiversity monitoring protocols strongly improved from 2009 onwards, we processed the data of the entire set of BDM plots recorded between the years 2009 and 2013. The data consisted of a matrix of documented presences and absences of all occurring vascular plant species per BDM plot. This allowed us to calculate species richness per BDM plot across Switzerland by summing the amount of species present in each plot. To assure that solely grassland species were assessed, we filtered nongrassland species from our data. Nongrassland species included shade‐tolerant species (below 10% relative illuminance), as well as species that inhabit forests, wetlands, and rocks. We based this on the characterization of all vascular plant species of Switzerland by the Flora indicativa of Landolt et al. (2010).

### 2.2 Functional trait data

We assembled plant trait data of both the Flora indicativa of Landolt et al. (2010), covering the entire Swiss Flora, as well as the Plant Trait Database (TRY) of 2016 (request no. 2256). The TRY database provides a global catalogue of curated plant traits (Kattge et al., 2011, 2020). We filtered the TRY database for grassland species using the list of grassland species created earlier (Kattge et al., 2020; Landolt et al., 2010). The TRY database included 60.2% of the grassland species of the Landolt database (2,438 species). We used specific leaf area (SLA) data of the TRY database in our analyses, as this trait allowed us to distinguish fast‐growing competitive species from slow‐growing stress‐tolerant ones (Díaz et al., 2015). Besides this, we chose to assess the influence of the nutrient preference (NPref) of grassland species, for which we derived data from the Flora indicativa of Landolt et al. (2010). The nutrient preference indicator of the Flora indicative is an ecological indicator, also commonly called EIV‐N or nutrient value N. The indicator is based on expert opinion as well as literature (Landolt et al., 2010). It is an ordinal variable consisting of 5 classes, representing a gradient from nutrient‐poor soils (1) to nutrient‐rich soils (5), mainly nitrogen (Landolt et al., 2010). Both SLA and NPref are related to productivity (Lavorel & Garnier, 2002; Peter, Gigon, Edwards, & Luscher, 2009). In each BDM plot, we assessed the following trait variables: mean NPref and SLA, the functional diversity of NPref and SLA, and the functional evenness of NPref and SLA. To calculate the mean of NPref and SLA, we used the function “mean” of the R‐package “base,” while we used the function “dbFD” of R‐package “FD” to calculate functional diversity and evenness. The “mean” function calculates the arithmetic mean, while the “dbFD” function calculates functional richness (FRic), functional evenness (Feve), and functional divergence (FDiv), as well functional dispersion (FDis), Rao's quadratic entropy (FraoQ), a posteriori functional group richness (FGR), and the community‐level weighted means of trait values (CWM) (Botta‐Dukát, 2005; Laliberté & Legendre, 2010; Petchey & Gaston, 2006; Villéger, Mason, & Mouillot, 2008). In this study, we use functional evenness, Rao's quadratic entropy, and the arithmetic mean because the BDM program only provides presence–absence data and no abundance data (BDM, 2014).

### 2.3 Climate data

Besides the effect of functional traits on productivity, we assessed the relative effect of the climatic variables temperature and precipitation. MeteoSwiss, the federal office of meteorology and climatology, provides data on temperature and precipitation for weather stations across Switzerland (MeteoSwiss, 2016a, 2016b). We used mean temperature and the sum of precipitation for each year for the growing season (April to September) throughout Switzerland from Daymet interpolated climate data at a 100‐m resolution and aggregated to 1‐km resolution to match our biodiversity data (Thornton, Running, & White, 1997). We used the spatial representation of the average temperature and the sum of precipitation over the growing season in Switzerland per year to extract temperature and precipitation values per BDM plot per year. Because temperature is highly linearly correlated with elevation in Switzerland (*R* ^2^ = .9457, *p*‐value < .005), we did not include elevation data in our analysis.

### 2.4 Grassland productivity data

**FIGURE 2:**
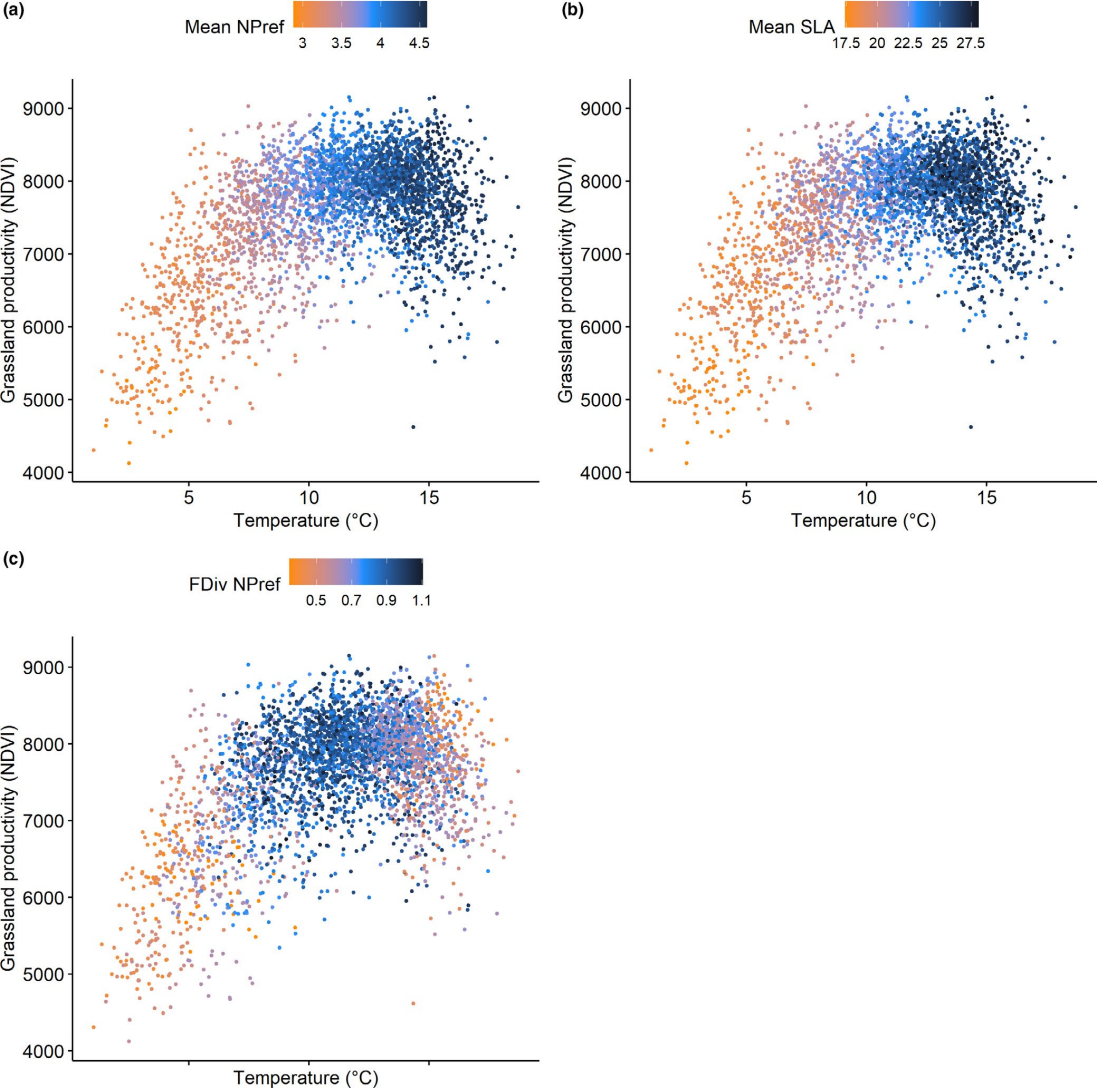
Productivity (NDVI) against temperature for the years 2009–2013 with the values of mean nutrient preference (mean NPref), mean specific leaf area (mean SLA) (mm^2^/mg), and the functional diversity of nutrient preference (FdivNPref) plotted using a color gradient from orange (low values) to dark blue (high values). It can be observed that productivity values increase with temperature until temperatures of approximately 13°C after which productivity drops. This could potentially be related to a loss in functional diversity of nutrient preference, which reduces niche complementarity (Hector et al., 1999)

We used the Normalized Difference Vegetation Index (NDVI) data as a proxy for the productivity of grasslands (Gandhi, Parthiban, Thummalu, & Christy, 2015; Pettorelli et al., 2005). We computed median NDVI values across Switzerland in a 30‐m resolution from a cloud‐free Landsat time series for the duration of the growing season (April‐September, four images per growing season) for the years 2001 to 2013. We used SWISSIMAGEs (compiled ortho‐photos forming a mosaic of Switzerland) from the Federal Office of Topography to manually distinguish grasslands from other land‐cover types (Swisstopo, 2015). Before we initiated our analyses, we assessed our assumption that the variation in productivity between BDM plots is higher than the variation in productivity within BDM plots. With a median of 430.62 of differences in NDVI between BDM plots and a median of 316.07 of differences in NDVI within BDM plots, this was the case. We, furthermore, tested the saturation of NDVI values (Appendix S1, Figure 2) and observed no saturation. We discarded all Landsat pixels (30‐by‐30 m) that were not associated with grassland within each 1‐by‐1 km BDM plot by hand. Of all 474 plots, 197 BDM plots did not contain more than 10% grassland. These plots were excluded, leaving 277 BDM plots for productivity assessment. For these 277 plots, we extracted the median NDVI values (NDVI is hereafter referred to as productivity) of all grassland points per year, that is, for 2001 to 2013 (four images per growing season). These values were subsequently averaged for each plot. They were also used to determine the temporal variance and coefficient of variance in productivity per BDM plot. The coefficient of variance was calculated as the standard deviation of productivity per BDM plot divided by the mean productivity per BDM plot.

### 2.5 Data organization

In total, we created three dataframes. One dataframe included total grassland productivity (values for each assessed year), one temporal variance in productivity (over all assessed years) and one the coefficient of variance in productivity (over all assessed years) as a response variable. Each dataframe, furthermore, included mean temperature and sum of precipitation (April to September), species richness and functional trait data per BDM plot as explanatory variables. Functional trait variables included mean NPref and SLA, the functional diversity of NPref and SLA, and the functional evenness of NPref and SLA. We used Rao's quadratic entropy as a measure of functional diversity and referred to this with FDiv. The dataframe including total productivity has varying values of productivity, temperature, and precipitation per year but fixed values for species richness and functional traits. The dataframes including temporal variance and the coefficient of variance in productivity have fixed values for temporal variance and the coefficient of variance in productivity, variance in temperature and variance in precipitation per year, as well as fixed values for species richness and functional traits. This allowed us to test the effect of climate, species richness, and plant trait composition on grassland productivity and temporal variation in grassland productivity across Switzerland.

### 2.6 Statistical analyses of productivity

We built linear mixed‐effects models (LMMs), using the function “lmer” of the R‐package “lme4” to test the effect of climate, species richness and plant trait composition on grassland productivity. LMMs contain both fixed effects and random effects (Nakagawa & Schielzeth, 2013; Vonesh, Chinchilli, & Pu, 1996). We chose LMMs for our analyses because of the strong heterogeneity in spatial and temporal scales in our data, which may influence the results. Unlike general linear models, LMMs account for this unobserved heterogeneity (Nakagawa & Schielzeth, 2013; Vonesh et al., 1996). All models included mean temperature and the sum of precipitation over the growing season (April to September) corresponding to the year of observation, as well as species richness or one specific plant trait variable. All variables were scaled using the function “scale” of the R‐package “base,” which centered and scaled our data to allow for comparison. We added the BDM plots and years as random factors in the models to exclude their potential effects on the results. Because we observed quadratic relationships between productivity and temperature, as well as between productivity and mean NPref, mean SLA and functional diversity of NPref, we added a quadratic term in addition to a linear term for these variables in the model.

A variance partitioning analysis was carried out to determine the relative contribution of species richness, individual trait variables, and the combined impact of temperature and precipitation on the total productivity of grasslands in Switzerland. As input for every variance partitioning analysis with grassland productivity as a response variable, we used three linear mixed‐effect models that included: (a) climatic variables and species richness or one trait variable, (b) climatic variables alone, or (c) species richness or one trait variable alone as explanatory variables. We calculated the R‐squared of all three models, using the function “r.squaredGLMM” of the R‐package “MuMIn,” which was also used for the variance partitioning analysis. The “r.squaredGLMM” function calculates both the conditional and marginal coefficient of determination. Marginal R‐squared concerns the variance explained by the fixed factors in the model, while the conditional R‐squared concerns variance explained by both fixed and random factors (Nakagawa & Schielzeth, 2013; Vonesh et al., 1996). We were, therefore, able to derive the total R‐squared of the linear mixed‐effect model, the R‐squared of the climatic variables and species richness or the plant trait variable together, the R‐squared of the climatic variables, the R‐squared of species richness or the plant trait variable, and the residuals (unexplained variance). We, furthermore, tested the significances of temperature (using ANOVA), precipitation, species richness, and plant traits, using the function “ANOVA” of the R‐package “car.”

### 2.7 Statistical analyses of the variance in productivity

General linear models were built to test the contribution of species richness, plant trait composition, and climatic variables on the temporal variance and the variance coefficient in productivity of grasslands across Switzerland, using the function “lm” of the R‐package “stats.”

We build general linear models because we accounted for the effect of both years and plots by calculating the variance in productivity and climatic variables between 2009 and 2013. All the models included the temporal variance or coefficient of variance in temperature and precipitation as climatic variables, and species richness or one specific trait variable as explanatory variables. To account for a normality deviation observed in both the raw data and residuals, the variance in productivity was log‐transformed. All variables were scaled using the function “scale” of the R‐package “base.” Because we observed quadratic relationships between the variance in productivity and mean NPref, as well as mean SLA, we added a quadratic term in addition to a linear term for these variables in the model. As for productivity, a variance partitioning analysis was carried out to determine the relative contribution of species richness, individual trait variables, and the combined effects of the variance and coefficient of variance of temperature and precipitation on the variance and coefficient of variance in productivity of grassland.

### 2.8 Model averaging

Using a model averaging approach, we determined the model that most consistently explained grassland productivity, using the function “AICc” of the R‐package “MuMIn”. AICc (Akaike Information Criterion for small sample size) calculated the out‐of‐sample prediction error of models for every combination of the explanatory variables provided. In total, we included all climatic and biodiversity variables in the model to understand which variables are contributing most to grassland productivity.

## 3 RESULTS

### 3.1 Relationship between productivity and biodiversity or functional trait composition

We found that temperature and, to a lesser extent, precipitation were positively associated with grassland productivity at the landscape‐scale, jointly explaining 19.8% of the variation in productivity (Table 1). Species richness was negatively associated with productivity, adding 2.4% explanatory power to a linear mixed‐effect model that includes climatic variables (i.e., temperature and precipitation) (Table 1). Mean plant nutrient preference (NPref), mean specific leaf area (SLA), and the functional diversity of NPref were positively associated to grassland productivity (Figure 2, Table 1). The full models including climate variables and either mean NPref, mean SLA or the functional diversity of NPref, explained 44.6%, 38.3%, and 30.8% of the variation in productivity respectively (Table 1). Independently, mean NPref, mean SLA, and functional diversity of NPref increased the explanatory power of the climatic linear mixed‐effect model with an additional 24.8%, 18.6%, and 11.0%, respectively. When all variables were considered, the model that explains grassland productivity best was a model that includes the evenness of NPref (Feve NPref), the functional diversity of NPref (FDiv NPref), mean NPref, mean SLA, and temperature (Appendix S2). The highest mean NPref and SLA values are located in intensive agricultural areas in the north of Switzerland. A relatively low functional diversity of NPref can be observed in intensively managed agricultural areas in the lowlands of Switzerland.

**TABLE 1:** Statistics of mixed models that include total productivity (NDVI) over the years 2009–2013 as a response variable and both the climatic variables temperature and precipitation (CV) as well as one species richness or trait variable as explanatory variables (lmer(NDVI (*response*) ~ CV (*explanatory*) + Species richness/Trait (*explanatory*) + (1|Years) (*random effect*) + (1|BDMplots) (*random effect*))

Independent linear mixed‐effects model variables	*p*‐value for temperature	*p*‐value for precipitation	*p*‐value for grassland species richness or trait	*R* ^2^ Total	*R* ^2^ Climatic and biodiversity variables (joint effect)	*R* ^2^ Climatic variables	*R* ^2^ Biodiversity variable	Residuals
Climatic variables	3.26E−25^***^	7.84E−04^***^	NA	.198	NA	NA	NA	0.802
Climatic variables + Species richness	2.39E−21^***^	.001^**^	.002^**^	.221	.037	.161	.024	0.779
Climatic variables + Mean NPref	.008^**^	.003^**^	2.11E−32^***^	.446	.182	.016	.248	0.554
Climatic variables + Mean SLA	6.88E−22^***^	.001^**^	4.89E−22^***^	.383	.176	.022	.186	0.617
Climatic variables + Fdiv NPref	3.48E−18^***^	.002^**^	4.10E−11^***^	.308	.040	.158	.110	0.692
Climatic variables + Fdiv SLA	4.72E−28^***^	.002^**^	.0004^***^	.228	.064	.134	.031	0.772
Climatic variables + Feve NPref	3.14E−28^***^	.003^**^	9.91E−10^***^	.295	−.004	.202	.097	0.705
Climatic variables + Feve SLA	6.88E−22^***^	.001^**^	.0004^***^	.230	−.019	.217	.032	0.770

Note

Each row in the table includes a summary of one linear model, including the significance of the effect of each variable in the model on the total productivity (*p*‐value), and the contribution of each variable to the goodness of fit of the model (*R* ^2^), as well as the contribution of all variables together on the goodness of fit of the model. Climatic variables = Temperature + Precipitation. Biodiversity variables = plant trait variable/species richness. Mean NPref = the mean of nutrient preference (NPref) of occurring grassland species per BDM plot, Mean SLA = the mean of specific leaf area (SLA) of occurring grassland species per BDM plot, Fdiv = functional diversity of NPref or SLA of grassland species per BDM plot, Feve = functional evenness of NPref or SLA per BDM plot. We quadrated the traits mean NPref, mean SLA, Fdiv NPref, Feve NPref, and NPref difference (poly(trait, 2)). Statistics: (a) “r.squaredGLMM” function of the R‐package “MuMin,” (b) “ANOVA” function of the R‐package “car.” The significance of an effect of a variable in each linear model on grassland productivity is highlighted with * for *p*‐values < .05, ** for *p*‐values < .005, and *** for *p*‐values < .0005.

### 3.2 Relationship between variance in productivity and biodiversity or functional trait composition

Compared with total productivity, both the temporal coefficient of variance and the variance in productivity were relatively poorly explained by the models that include climatic and functional trait variables (Tables 2 and 3). A model that solely includes climatic variables explained 1.3% and 1.7% of the temporal coefficient of variance and variance in productivity, respectively. Functional diversity of NPref only added 3.0% and 3.1% of explanatory power to the temporal coefficient of variance and variance models, respectively, while mean SLA only added 1.5% and 2.4% of explanatory power (Tables 2 and 3). Species richness is positively related to the temporal coefficient of variance and variance in productivity and added 5.4% and 5.1% of explanatory power to the climatic models, respectively. Only mean NPref showed a higher signal and was negatively associated to the temporal coefficient of variance in productivity, explaining 5.8% and 7.0% of the temporal coefficient of variance and variance in productivity respectively, while accounting for variance in temperature and precipitation (Tables 2 and 3).

**TABLE 2:** Statistics of linear models that include the coefficient of variance of productivity (NDVI) as a response variable and both the coefficient variance of both the climatic variables temperature and precipitation (CV) as well as one species richness or trait variable as explanatory variables (lm(NDVI (*response*) ~ CV (*explanatory*) + Species richness/Trait (*explanatory*)))

Independent linear mixed‐effects model variables	*p*‐value for temperature	*p*‐value for precipitation	*p*‐value for grassland species richness or trait	*R* ^2^ Total	*R* ^2^ Climatic and biodiversity variables (joint effect)	*R* ^2^ Climatic variables	*R* ^2^ Biodiversity variable	Residuals
Climatic variables	.769	.129	NA	.013	NA	NA	NA	0.987
Climatic variables + Species richness	.436	.073	.001^**^	.067	−.007	.020	.054	0.933
Climatic variables + Mean NPref	.332	.141	.004^**^	.071	−.005	.018	.058	0.929
Climatic variables + Mean SLA	.676	.215	.243	.028	.002	.010	.015	0.972
Climatic variables + Fdiv NPref	.803	.082	.018^*^	.042	−.003	.016	.030	0.958
Climatic variables + Fdiv SLA	.662	.127	.257	.019	−.000	.012	.007	0.981
Climatic variables + Feve NPref	.758	.041^*^	.007^**^	.051	−.010	.022	.038	0.949
Climatic variables + Feve SLA	.656	.106	.003 ^**^	.059	−.002	.015	.047	0.941

Note

Each row in the table includes a summary of one linear model, including the significance of the effect of each variable in the model on the coefficient of variance in productivity (*p*‐value), and the contribution of each variable to the goodness of fit of the model (*R* ^2^), as well as the contribution of all variables together on the goodness of fit of the model. Climatic variables = Temperature + Precipitation. Biodiversity variables = plant trait variable/species richness. Mean NPref = the mean of nutrient preference (NPref) of occurring grassland species per BDM plot, Mean SLA = the mean of specific leaf area (SLA) of occurring grassland species per BDM plot, Fdiv = functional diversity of NPref or SLA of grassland species per BDM plot, Feve = functional evenness of NPref or SLA per BDM plot. Statistics: (a) “r.squaredGLMM” function of the R‐package “MuMin”, (b) “ANOVA” function of the R‐package “car.” The significance of an effect of a variable in each linear model on grassland productivity is highlighted with * for *p*‐values < .05, ** for *p*‐values < .005, and *** for *p*‐values < .0005.

**TABLE 3:** Statistics of linear models that include the variance of productivity (NDVI) as a response variable, and both the variance of both the climatic variables temperature and precipitation (CV) as well as one species richness or trait variable as explanatory variables (lm(NDVI (*response*) ~ CV (*explanatory*) + Species richness/Trait (*explanatory*)))

Independent linear mixed‐effects model variables	*p*‐value for temperature	*p*‐value for precipitation	*p*‐value for grassland species richness or trait	*R* ^2^ Total	*R* ^2^ Climatic and biodiversity variables (joint effect)	*R* ^2^ Climatic variables	*R* ^2^ Biodiversity variable	Residuals
Climatic variables	.682	.091	NA	.017	NA	NA	NA	0.983
Climatic variables + Species richness	.178	.058	.002^**^	.068	−.012	.028	.051	0.931
Climatic variables + Mean NPref	.082	.124	.001^**^	.087	−.013	.030	.070	0.913
Climatic variables + Mean SLA	.247	.180	.102	.041	−.001	.018	.024	0.959
Climatic variables + Fdiv NPref	.384	.051	.016^*^	.047	−.007	.024	.031	0.953
Climatic variables + Fdiv SLA	.852	.084	.341	.022	.000	.016	.005	0.978
Climatic variables + Feve NPref	.384	.032^*^	.016^*^	.047	−.011	.028	.031	0.953
Climatic variables + Feve SLA	.270	.072	.001^**^	.072	−.006	.023	.055	0.928

Note

Each row in the table includes a summary of one linear model, including the significance of the effect of each variable in the model on the variance in productivity (*p*‐value), and the contribution of each variable to the goodness of fit of the model (R^2^), as well as the contribution of all variables together on the goodness of fit of the model. Climate = Temperature + Precipitation. Biodiversity variables = plant trait variable/species richness. Mean NPref = the mean of nutrient preference (NPref) of occurring grassland species per BDM plot, Mean SLA = the mean of specific leaf area (SLA) of occurring grassland species per BDM plot, Fdiv = functional diversity of NPref or SLA of grassland species per BDM plot, Feve = functional evenness of NPref or SLA per BDM plot. Statistics: (a) “r.squaredGLMM” function of the R‐package “MuMin,” (b) “ANOVA” function of the R‐package “car.” The significance of an effect of a variable in each linear model on grassland productivity is highlighted with * for *p*‐values < .05, ** for *p*‐values < .005, and *** for *p*‐values < .0005.

## 4 DISCUSSION

### 4.1 Relationship between productivity and biodiversity or functional trait composition

Our landscape‐scale analyses indicate that biodiversity variables, in particular trait variables, help explain productivity patterns better than climatic variables alone. This is interesting given the large gradient of climatic variables (Figure 2). Mean SLA alone explains variation in productivity better than climatic variables. The strong effects of functional traits on productivity might result from historical effects or management practices, which affects the species pool and functional compositions of grasslands (Cadotte, Cavender‐Bares, Tilman, & Oakley, 2009; Chalmandrier et al., 2017). Hence, functional traits displayed by species may be used as predictors of niche complementarity (Díaz & Cabido, 2001). Overall, the effect of plant trait composition on grassland productivity found in our study is in agreement with experimental studies, which showed that the trait composition of grassland ecosystems is a stronger determinant of ecosystem processes than species richness (Cadotte et al., 2009; Tilman et al., 1997). The weak relationship between species richness and productivity may be explained by elevation gradients, which causes a simultaneous change of species richness and composition with the environment (Wohlgemuth, Nobis, Kienast, & Plattner, 2008), an effect that is usually not included in experimental field studies (Tilman, Reich, Knops, & Mielke, 2001).

**FIGURE 3:**
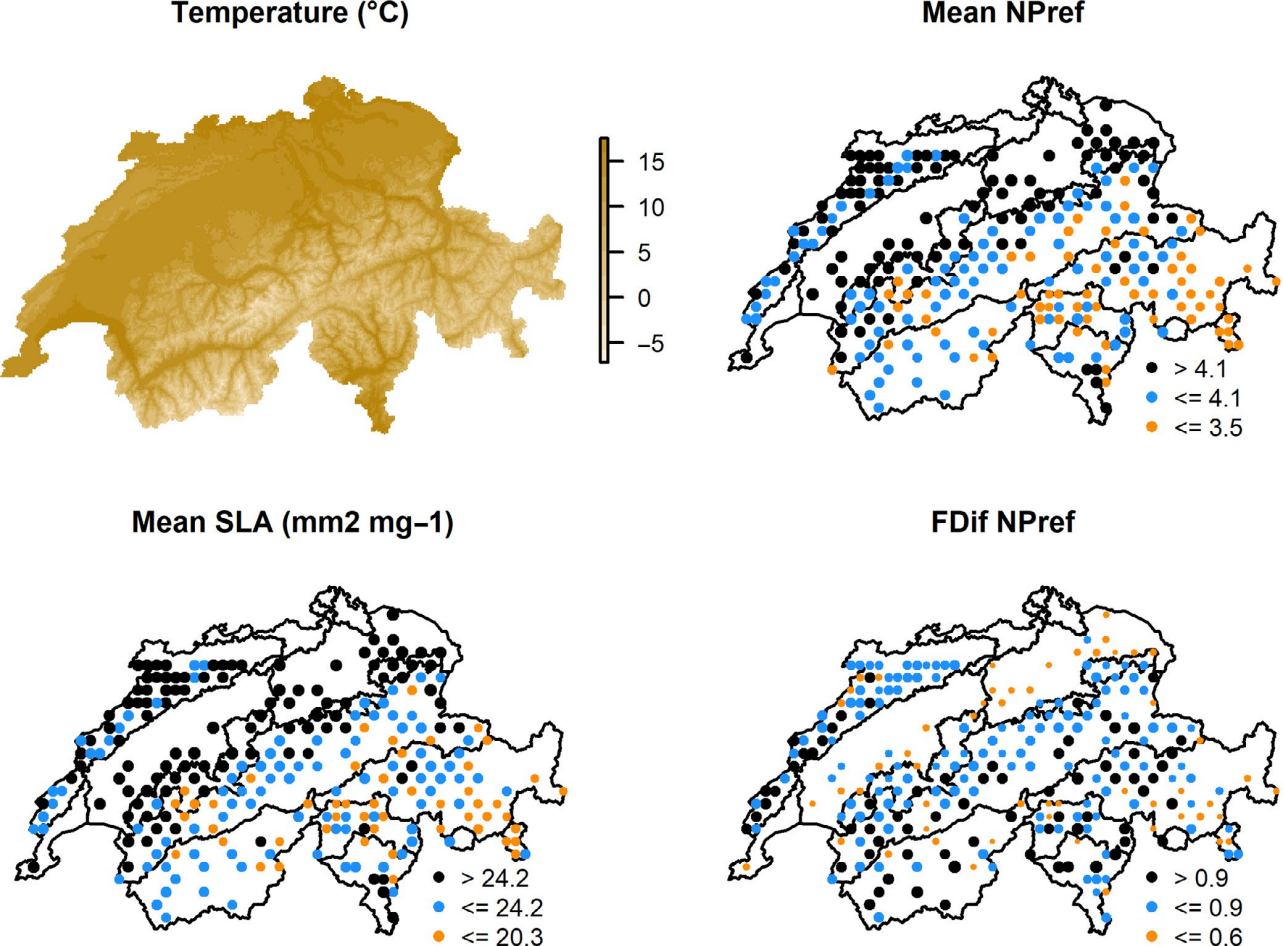
Maps of Switzerland on which temperature (°C), mean nutrient preference (NPref), mean specific leaf area (SLA) (mm^2^/mg), and the functional diversity of NPref (Fdiv NPref) is plotted per grassland‐harboring plot of the Biodiversity Monitoring Program (BDM) in Switzerland. The size of the dots increases with from the lowest to the highest values for mean nutrient preference (NPref), mean specific leaf area (SLA) (mm^2^/mg), and the functional diversity of NPref (Fdiv NPref). The highest mean NPref and SLA values are located in intensive agricultural areas in the north of Switzerland, which is likely due to fertilization and seed mix application (FSO, 2015). The relationship between productivity and mean NPref and mean SLA is furthermore influenced by elevation, since soils at higher elevation are more nutrient‐poor than soils at lower elevation (Peter et al., 2009). Therefore, solely stress‐tolerant species with a low SLA and NPref are able to grow at higher elevations, resulting in relatively low mean NPref and mean SLA values in the Swiss Alps (Körner, 1989). A relatively low functional diversity of NPref can be observed in intensively managed agricultural areas in the lowlands of Switzerland. This supports the hypothesis that a bias in the species pool toward more productive species occurs in these areas

The association between productivity and NPref, as well as SLA, can be explained by productivity enhancement through fertilization and seed mix application in agricultural areas (FSO, 2015). In managed grasslands, farmers tend to introduce plant species that are highly palatable for cattle (Salomon, Engström, Nilsdotter‐Linde, & Spörndly, 2019). Therefore, a direct or indirect selection of plant species with high SLA or plant species with preferences for rich soils may occur. In contrast, nutrient‐poor—but species‐rich—grasslands are less productive. Our analysis shows that highest mean NPref and SLA values are located in the relatively warm intensive agricultural areas in the north of Switzerland as fast‐growing species with a higher mean NPref and mean SLA outcompete more nutrient stress‐tolerant but slow‐growing species in conditions of high nutrient availability (FSO, 2015; Wang et al., 2007) (Figure 3). Grassland productivity is also related to climate, and we show that it is lower in the relatively colder areas of Switzerland located at high elevation (Figure 3). Moreover, some of the variance in our models was shared between climate and functional traits, which is likely due to changes in functional trait composition with climatic variables (Figure 2). Stress‐tolerant species with a low SLA and low NPref are more frequent at higher elevations, and their lower productivity can be explained by the slower growth of those species and the more stressful climate they occur in (Körner, 1989). Hence, at a landscape‐scale, our results indicate that a combination of climate and functional composition of grasslands influences productivity.

Our results contrast with the results of Oehri et al. (2017) and Grace et al. (2016). They show a positive relationship between species richness and productivity across the Swiss landscape. Oehri et al. (2017), however, include all ecosystems at once, while we focus specifically on grasslands. Besides this, Oehri et al. (2017) did not investigate functional traits and quadratic terms were not included in the statistical models, while the relationship between climate and productivity is not always linear (Whittaker 1970; Zhang & Wei, 2017). In our dataset, we observed a nonlinear effect between temperature and productivity. We assume that this effect results from a saturation of growth in the warmest, but also the driest regions of Switzerland. Moreover, although Oehri et al. (2017) based their analyses on the same plant diversity data as in our study (BDM), they analyzed the relationship of species richness on productivity across all ecosystems present in the BDM plots, whereas our study solely focusses on grassland productivity. The contrast between our study and the study of Grace et al. (2016) may be explained by the differences in scale (Grace et al., 2016 assessed effects of species richness on a global scale). Besides this, they did not assess the impact of functional traits; hence, it is unknown whether these affect productivity on a global scale. The effects of trait composition rather than species richness on productivity found in our study are in agreement with Winfree et al. (2015) who showed that not species richness but the presence of a few abundant species drives ecosystem functioning in natural ecosystems. Van der Plas (2019), furthermore, indicated that metrics of functional diversity are stronger predictors of ecosystem functioning than species richness in naturally assembled communities. This could explain why Grace et al. (2007) did not find a significant effect of biodiversity on productivity at a landscape‐scale when solely species richness was considered. The contrasting results among studies highlight the complexity of analyzing the effect of biodiversity on productivity in real landscape, due to the strong interrelation of variables. For instance, we observed a relatively higher species richness and larger variation in productivity in the alpine regions of Switzerland compared with the lowland regions.

Although productivity increases with temperature, a sudden decrease in productivity can be observed at temperatures above 13°C (Figure 2). Regions with average growing season temperatures above 13°C are situated in the intensive agricultural areas in the northern lowlands of Switzerland, where the highest values of mean NPref and mean SLA are found (Figure 3). With the expected global change‐induced temperature increase (IPCC, 2013), it is crucial to determine the response of grassland productivity of grassland communities around the world to prevent economic losses for livestock farmers. In addition, although precipitation in our study is momentarily relatively poorly related to productivity, the expected global change‐induced decrease in precipitation is projected to affect Switzerland in the near future (Meehl & Tebaldi, 2004). Overall, the drop in NDVI may be due the high‐intensity management that takes place in the agricultural regions in Switzerland with higher rates of cutting but further studies are necessary to confirm this. Future studies could assess the impact of management intensity on functional trait composition at a landscape level in Switzerland to better understand the drop in productivity above 13°C and whether this is related to biodiversity or other variables.

### 4.2 Relationship between variance in productivity and biodiversity or functional trait composition

The mean of NPref was negatively associated to the coefficient of variance in productivity, indicating that grasslands composed of species with affinities for high nutrient availability have both a higher and more stable productivity. Thus far, farmers have adopted the most efficient management strategy, but the low functional diversity in those grasslands may only be able to weakly buffer the effects of future climate change on fodder production. The observed weak but negative effect of functional diversity of NPref on variance is in agreement with the observation of several experimental studies showing that plant functional composition might modify the temporal stability of productivity due to a functional turnover and niche complementarity (Isbell, Polley, & Wilsey, 2009). Overall, compared with the total productivity, the variance in productivity is relatively poorly explained by the models and the results should be interpreted with caution. The poor explanation of variance in productivity by species richness and functional trait composition by our models contrasts with the consensus that more species are needed to ensure a stable delivery of ecosystem services upon spatiotemporal variations in weather and climate (Hooper et al., 2005). It is possible that nonpermanent grasslands were considered in our analyses, and turnover in the use of those land patches might blur the relationships. Artificial grasslands found in the warmest regions of Switzerland are cut four or five times a year in intensely management grasslands, which could result in an artificial reduction of grassland productivity in this region, and might explain the sudden decrease in productivity at temperatures of about 13°C. However, when assessing the variations in productivity per geographic region (Appendix S1, Figure 1), the mountain areas showed a higher variation than the lowland areas (Appendix S3, Figure 1). This indicates that the effects of management may be smaller than the effects of climatic variations in temperature and precipitation.

### 4.3 Limitations

Because this study relies on remote sensing, there are several limitations regarding the processed data, as well as the methodology. First, this study relies on 277 grassland‐harboring 1‐by‐1 km plots across Switzerland to derive conclusions about the entire country. Moreover, since the TRY data did not contain all grassland species of Switzerland, not all species are included in this study to calculate the functional composition of the plant communities. We found that the amount of missing species in the TRY database is relatively equally spread across the bioregions of Switzerland. Of the grassland species found in the BDM plots, above 60% were present in the TRY database for all biogeographical regions (Appendix S4, Figure 1). It is likely that subordinate species (i.e., species that never attain dominance but are found in most plots (Mariotte, 2014)) or rare species are missing from the list with relatively low mean SLA and mean NPref values. Because of more extreme environments, more subordinate species may be found in the high Alps. We did not find a strong skewness toward low mean SLA and mean NPref values, except for the mean NPref of the Southern and Eastern Alps (Appendix S5, Figures 10 and 12), indicating that the effects of extreme environments on the distribution of SLA and NPref values are limited. However, it could be that species with low mean SLA and mean NPref were excluded, which would explain the lack of skewness. When assessing the variance in mean NPref and mean SLA between biogeographical regions, we observed higher variance in the mountain regions (Appendix S6, Figures 1 and 2). This indicates that sufficient species with low mean NPref and mean SLA values are included in our sample to allow for large variations in mean NPref and mean SLA values across Switzerland. Based on this, we may assume that we take subordinate species into account in our analyses. Second, temperature and precipitation data were interpolated across Switzerland using climate data from Meteoswiss weather stations. This is more specific for temperature, since it is interpolated with elevation as a proxy, while mapping precipitation is a more challenging task. Third, the management intensity in the BDM plots is unknown. Therefore, it is uncertain how management intensity (e.g., irrigation, mowing, grazing, and fertilization) influences the results of this study, especially as irrigation of grassland could affect the statistical outcome of precipitation. Unfortunately, we did not find data on management intensity across Switzerland of a sufficiently low spatial resolution to assess the effect on productivity, species richness, or traits per BDM plot. Fourth, the BDM program solely records the occurrence of species and not their abundance (BDM, 2014). Taking species abundance into account would likely have improved our results because the most dominant species attribute most to the NDVI signal. Finally, the use of NDVI as a proxy for productivity is currently under debate, since NDVI is found to saturate at high biomass values (Santin‐Janin, Garel, Chapuis, & Pontier, 2009). However, our study assesses grasslands, which are relatively low in biomass compared with forests. Therefore, we do not expect to reach these high values and we, furthermore, did not find an indication of saturating of NDVI (Appendix S1, Figure 2). In addition, NDVI signals are influenced by repeated grassland mowing (Kolecka, Ginzler, Pazur, Price, & Verburg, 2018), which may have influenced our productivity estimations in agricultural areas of Switzerland.

## 5 CONCLUSION

The relationships between functional traits and grassland productivity found in our study indicate the importance of including functional trait variables to explain productivity on a landscape‐scale. The contrast between the results of our study and those of highly controlled experimental studies may be explained by the large landscape‐scale approach of our study compared with the generally small scale of diversity experiments. Unlike experimental studies, diversity at the landscape level is determined by multiple community assembly processes, such as variations in the natural species pool and environmental heterogeneity. Besides this, diversity in experimental studies is only slightly influenced by randomly occurring anthropogenic and climatic perturbations, while these processes exert a strong influence on diversity at a landscape level. This indicates the importance of a landscape‐scale assessment to determine the real‐world effects of biodiversity on productivity and other ecosystems services. The results of our study, furthermore, advocate for the inclusion of functional traits to enhance productivity in grasslands, which may secure resistance and resilience in fodder production. Our results show a strong bias in the species pool due to the indirect selection of the fastest‐growing and most nitrogen‐rich grassland species by farmers. However, both permanent grasslands and seed mixtures for artificial grasslands should be adapted by promoting plant trait compositions that may enhance resilience of grasslands to global change (e.g., drought events). This can be done by, for instance, increasing the diversity of species in seed mixes and reducing the amount of fertilizer applied to the land. More data are necessary to further unravel the real‐life relationships between biodiversity and productivity, as well as to understand the unexplained variance in productivity.

## CONFLICT OF INTEREST

The authors declare no competing interests.

## AUTHOR CONTRIBUTIONS

**Hanneke van't Veen:** Data curation (lead); formal analysis (lead); visualization (equal); writing – original draft (lead). **Loïc Chalmandrier:** Data curation (supporting); formal analysis (supporting); investigation (equal); methodology (equal); supervision (supporting); writing – review & editing (equal). **Nadine Sandau:** Writing – review & editing (equal). **Michael P. Nobis:** Resources (lead); writing – review & editing (equal). **Patrice Descombes:** Data curation (equal); formal analysis (equal); supervision (supporting); writing – review & editing (equal). **Achilleas Psomas:** Resources (equal); writing – review & editing (equal). **Yann Hautier:** Supervision (supporting); writing – review & editing (equal). **Loïc Pellissier:** Conceptualization (lead); data curation (equal); formal analysis (equal); funding acquisition (lead); supervision (lead); visualization (supporting); writing – review & editing (lead).

## Supporting information

Appendix S1‐1

Appendix S1‐2A

Appendix S1‐2B

Appendix S3‐1

Appendix S4‐1

Appendix S5‐1

Appendix S5‐3

Appendix S5‐4

Appendix S5‐5

Appendix S5‐6

Appendix S5‐7

Appendix S5‐8

Appendix S5‐9

Appendix S5‐10

Appendix S5‐11

Appendix S5‐12

Appendix S6‐1

Appendix S6‐2

Appendix S1‐S6

## Data Availability

All plant trait data are available on the TRY database. The Landsat data used are available on the Landsat portal. The species composition data will be provided upon publication of the manuscript, apart from the BDM data which are not allowed to publish according to signed data agreements.
