# Supplementary figures and images for: A landscape‐scale assessment of the relationship between grassland functioning, community diversity, and functional traits
Source: Ecol Evol. 2020 Aug 16;10(18):9906–19. doi: 10.1002/ece3.6650 (PMC7520175; doi:10.1002/ece3.6650)

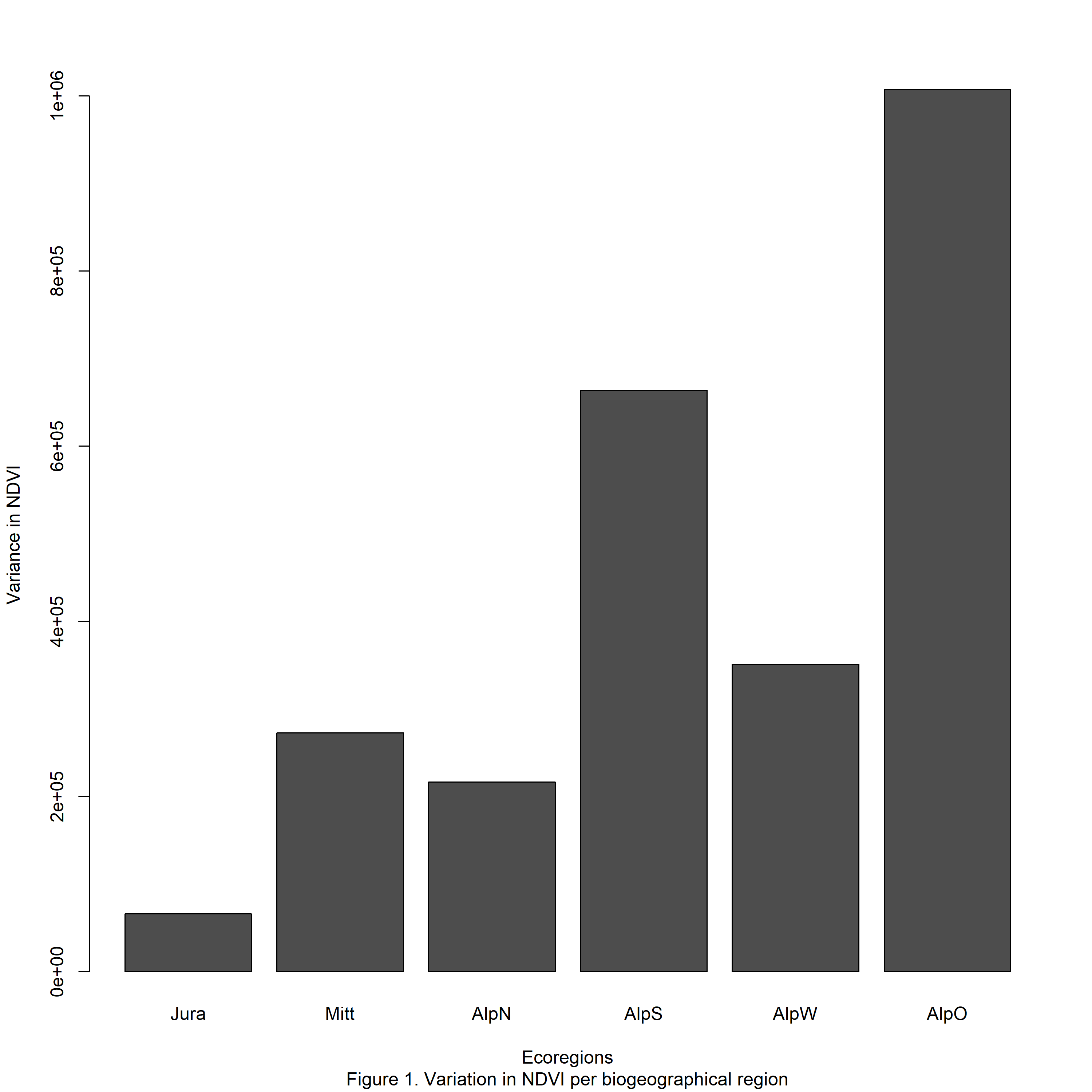

Supplement: Supplementary file 1 — Appendix S1‐1 [file ECE3-10-9906-s001.tiff]

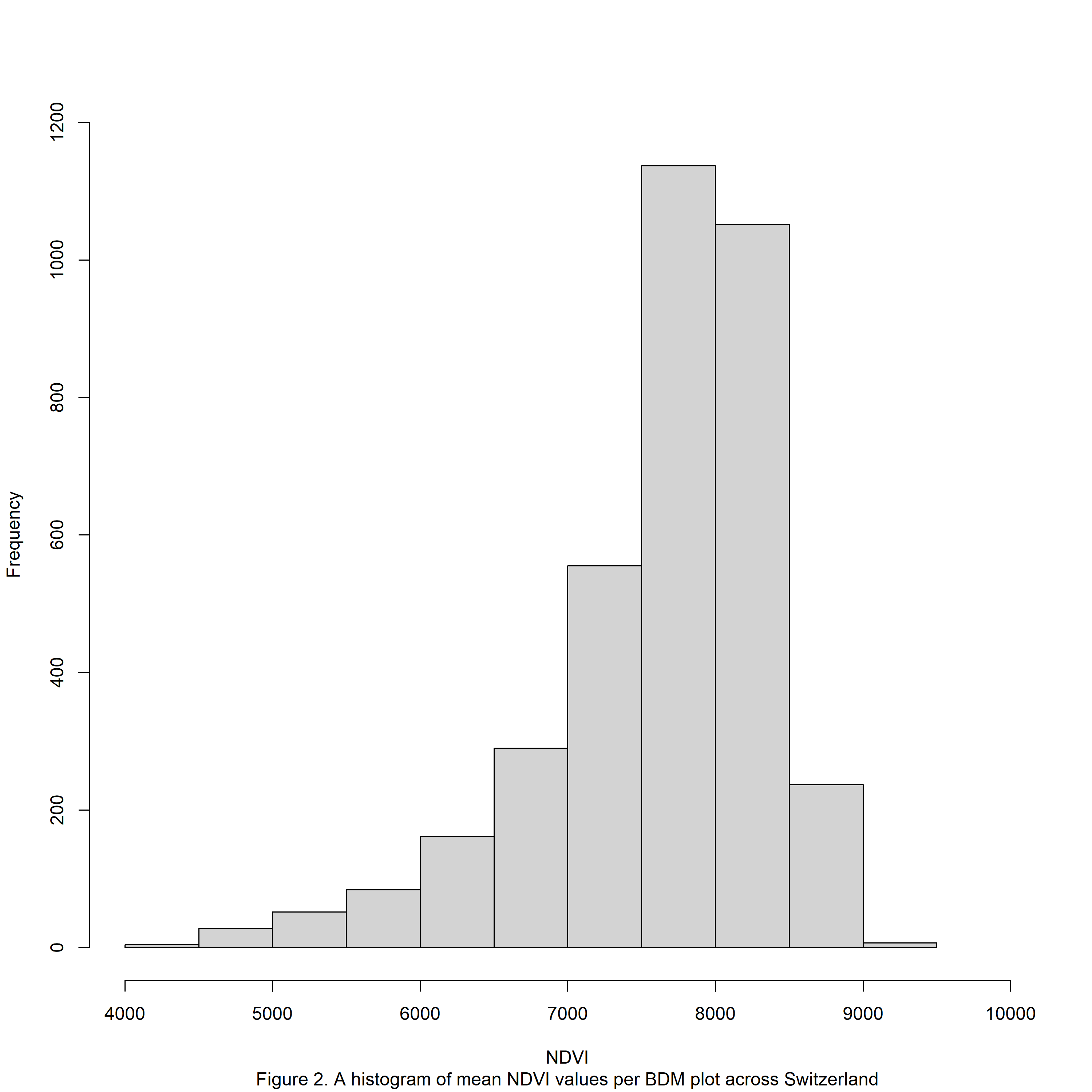

Supplement: Supplementary file 2 — Appendix S1‐2A [file ECE3-10-9906-s002.tiff]

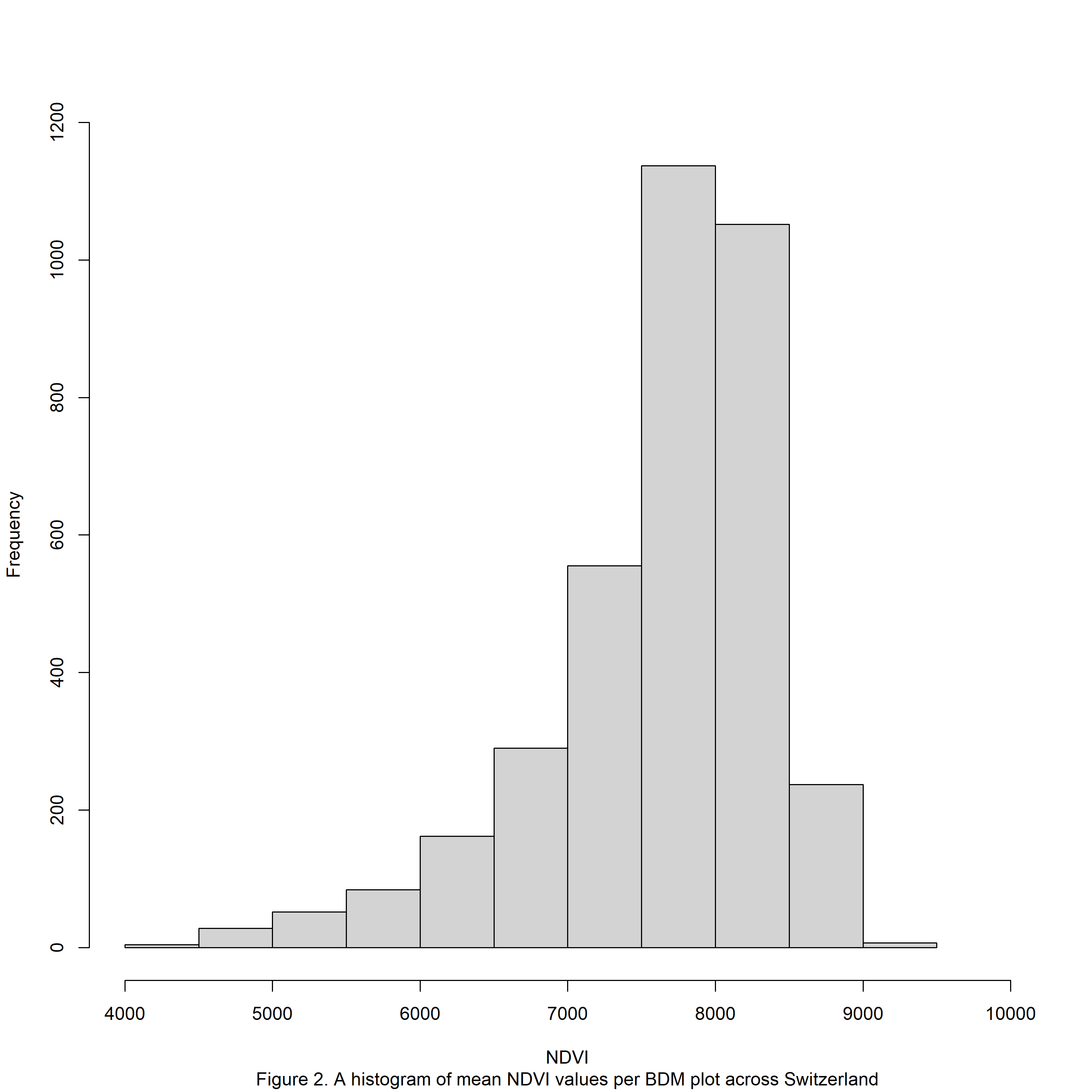

Supplement: Supplementary file 3 — Appendix S1‐2B [file ECE3-10-9906-s003.tiff]

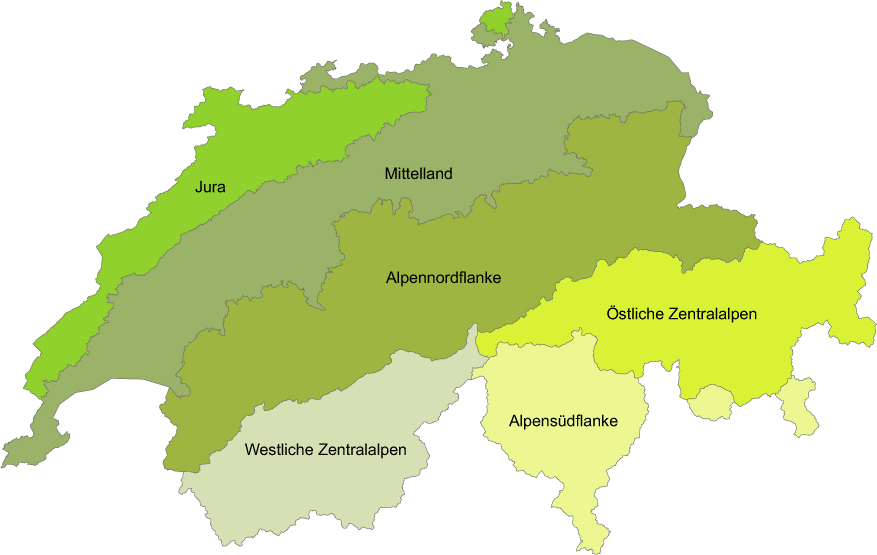

Supplement: Supplementary file 4 — Appendix S3‐1 [file ECE3-10-9906-s004.gif]

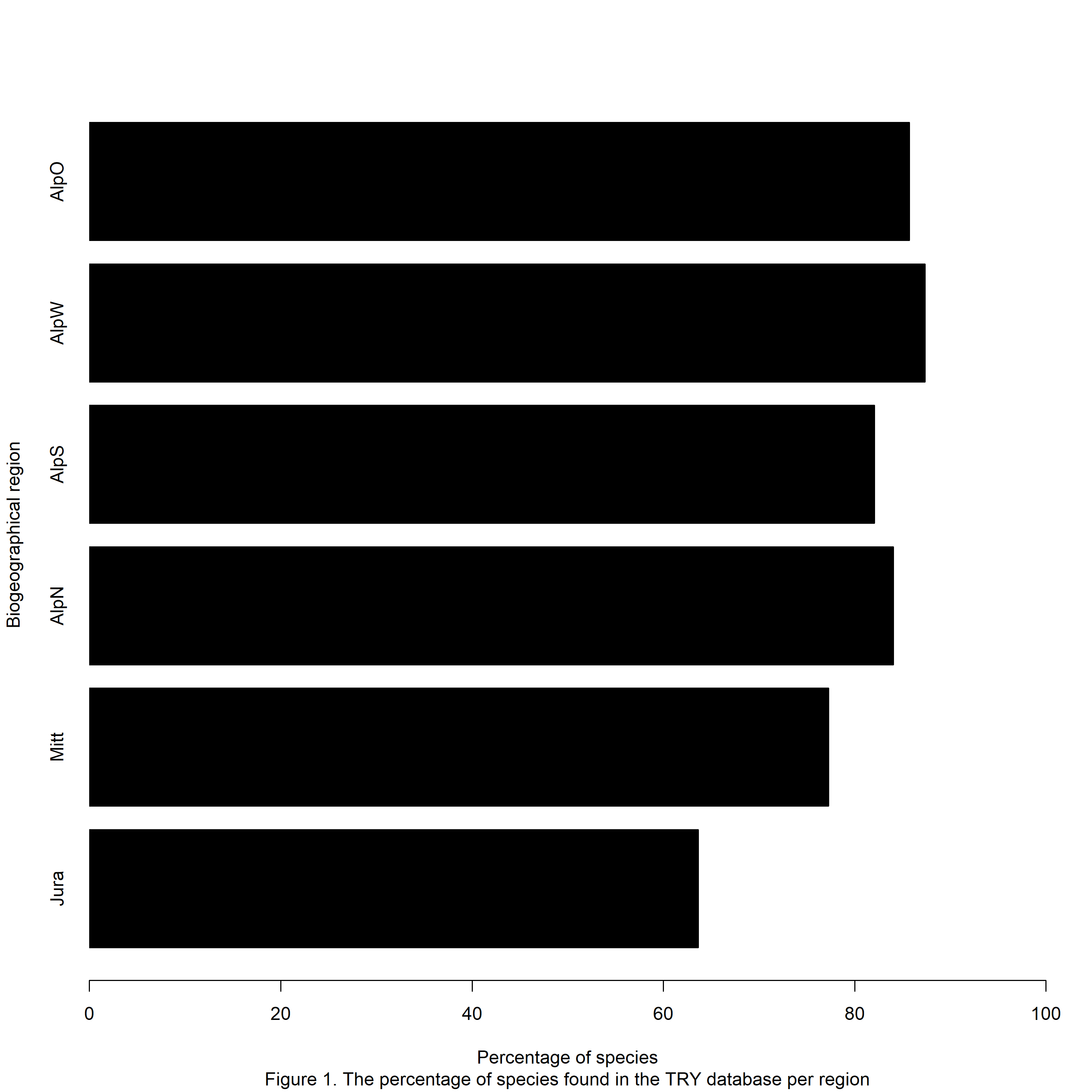

Supplement: Supplementary file 5 — Appendix S4‐1 [file ECE3-10-9906-s005.tiff]

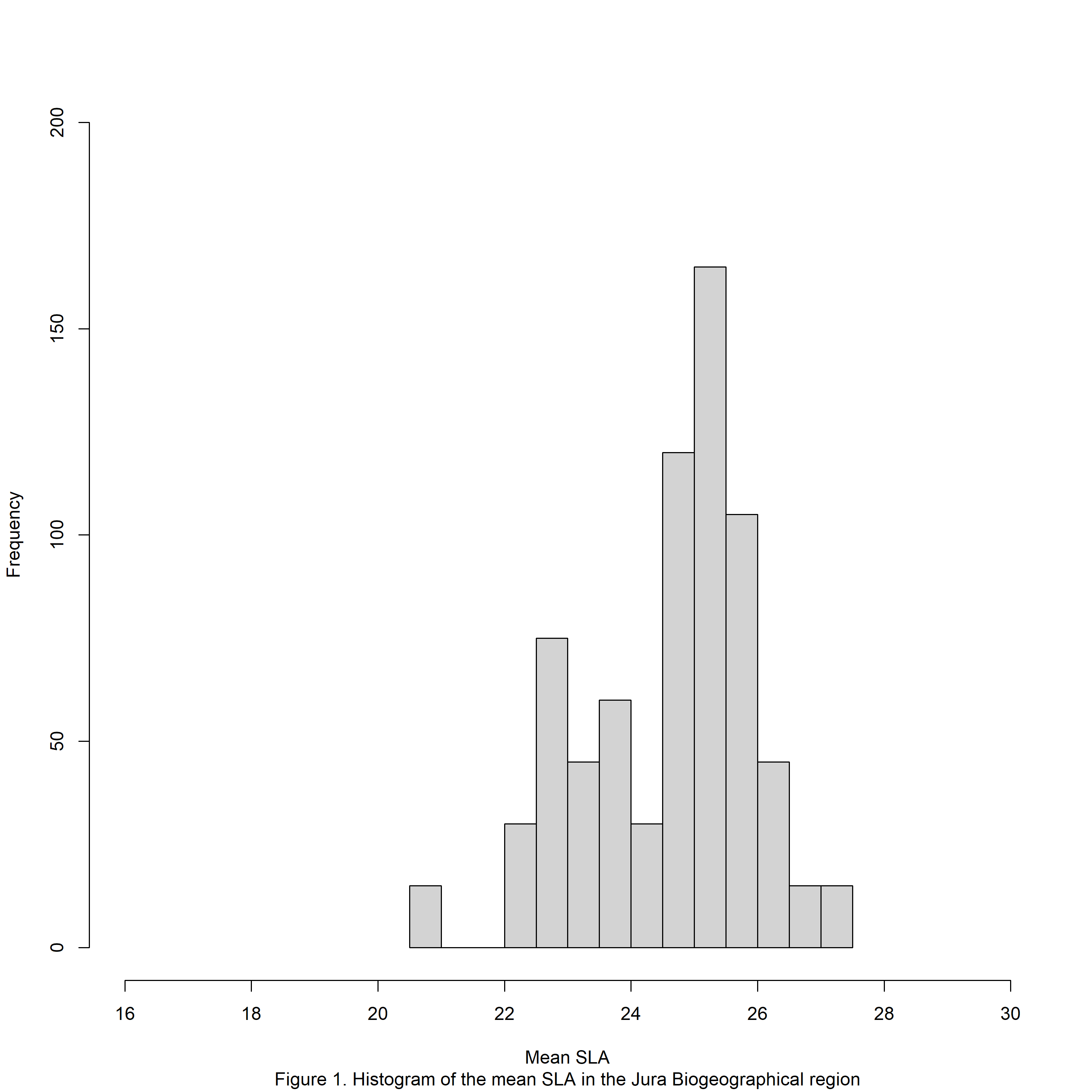

Supplement: Supplementary file 6 — Appendix S5‐1 [file ECE3-10-9906-s006.tiff]

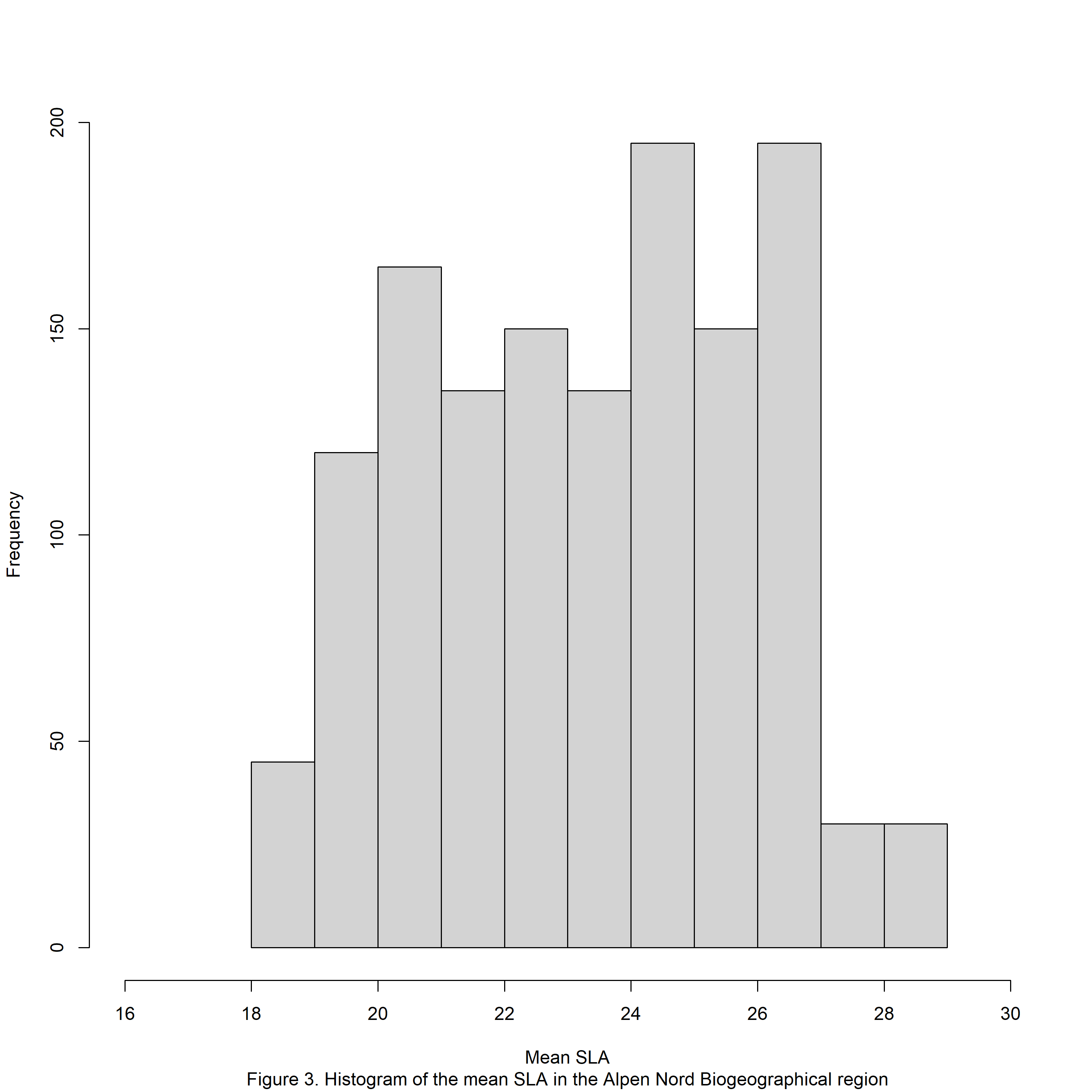

Supplement: Supplementary file 7 — Appendix S5‐3 [file ECE3-10-9906-s007.tiff]

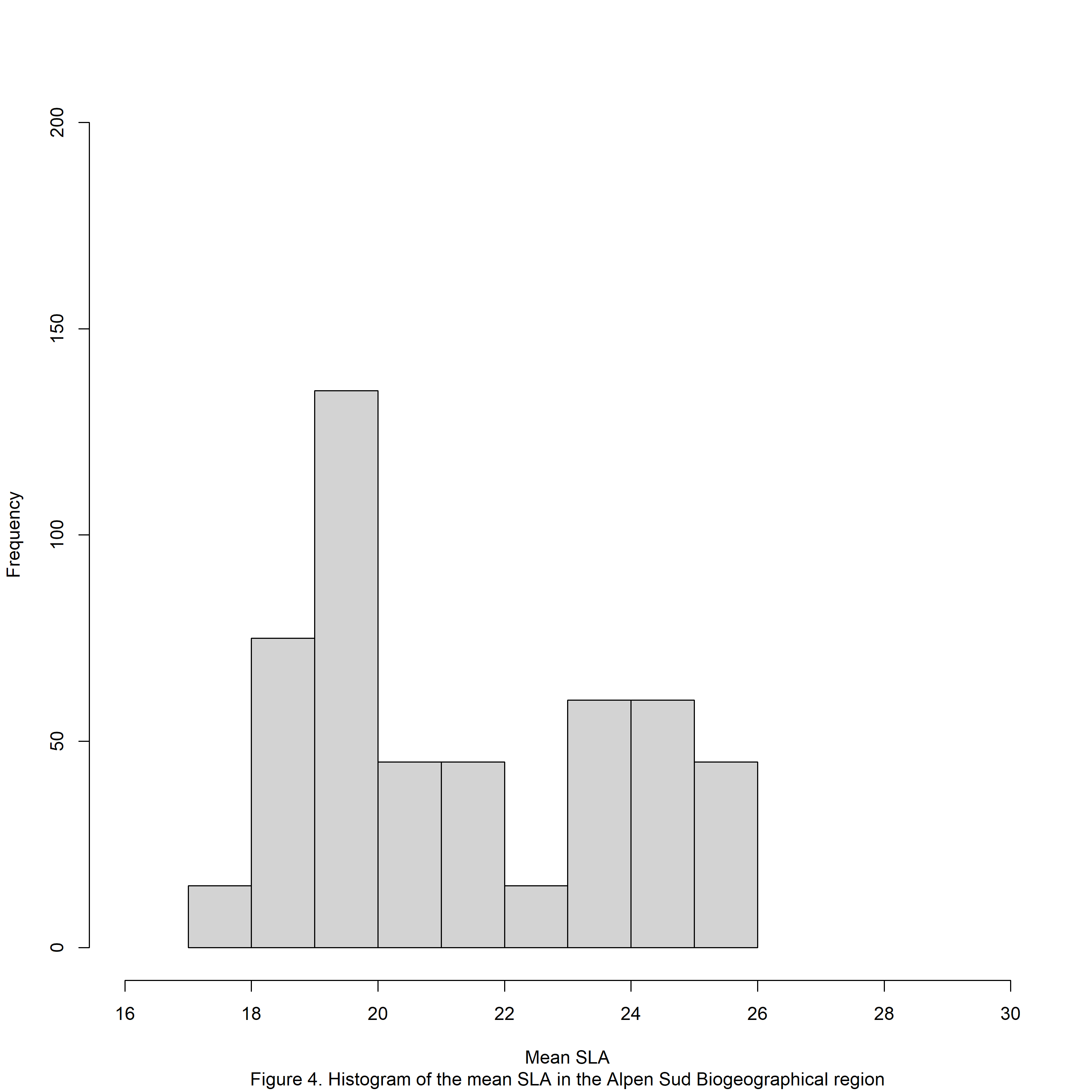

Supplement: Supplementary file 8 — Appendix S5‐4 [file ECE3-10-9906-s008.tiff]

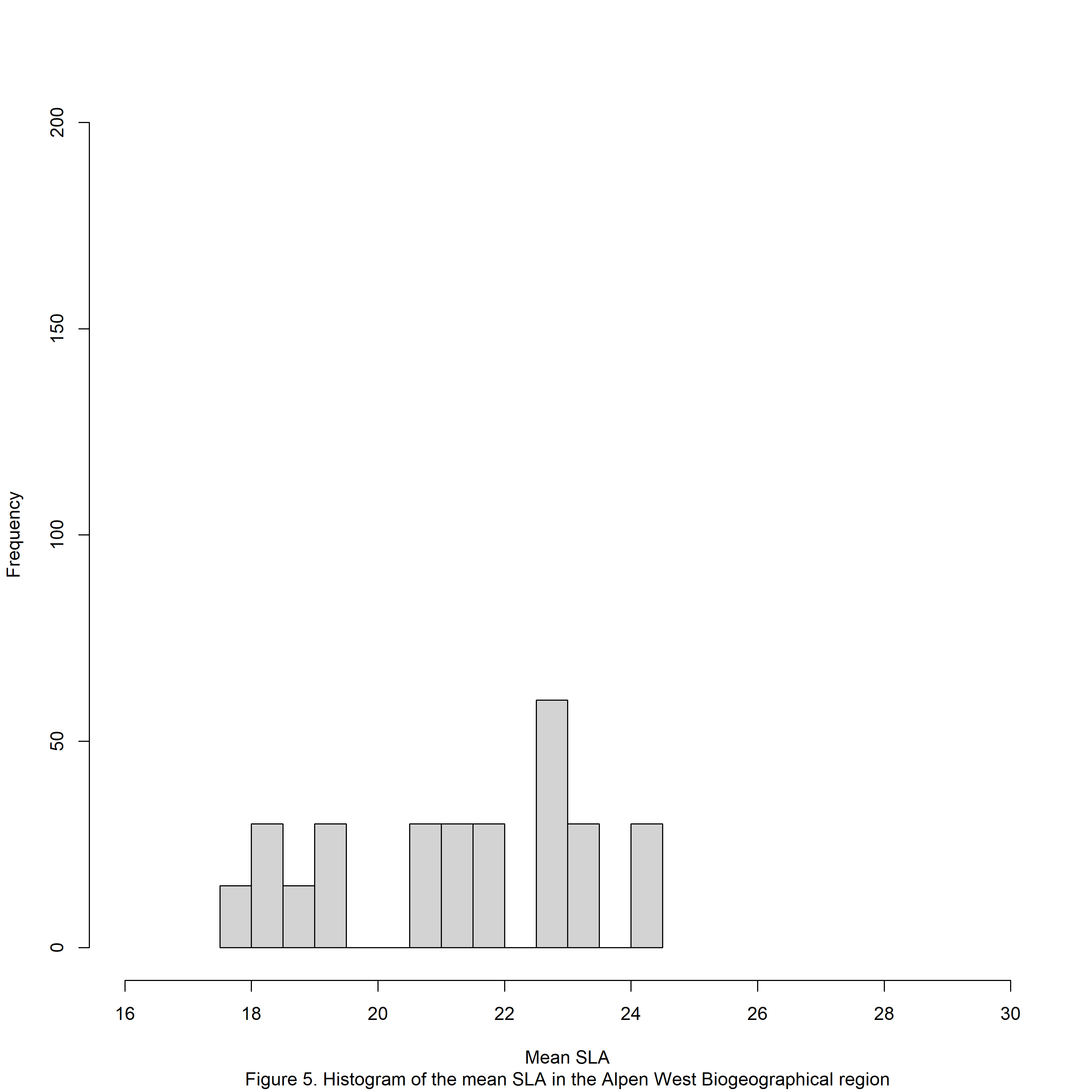

Supplement: Supplementary file 9 — Appendix S5‐5 [file ECE3-10-9906-s009.tiff]

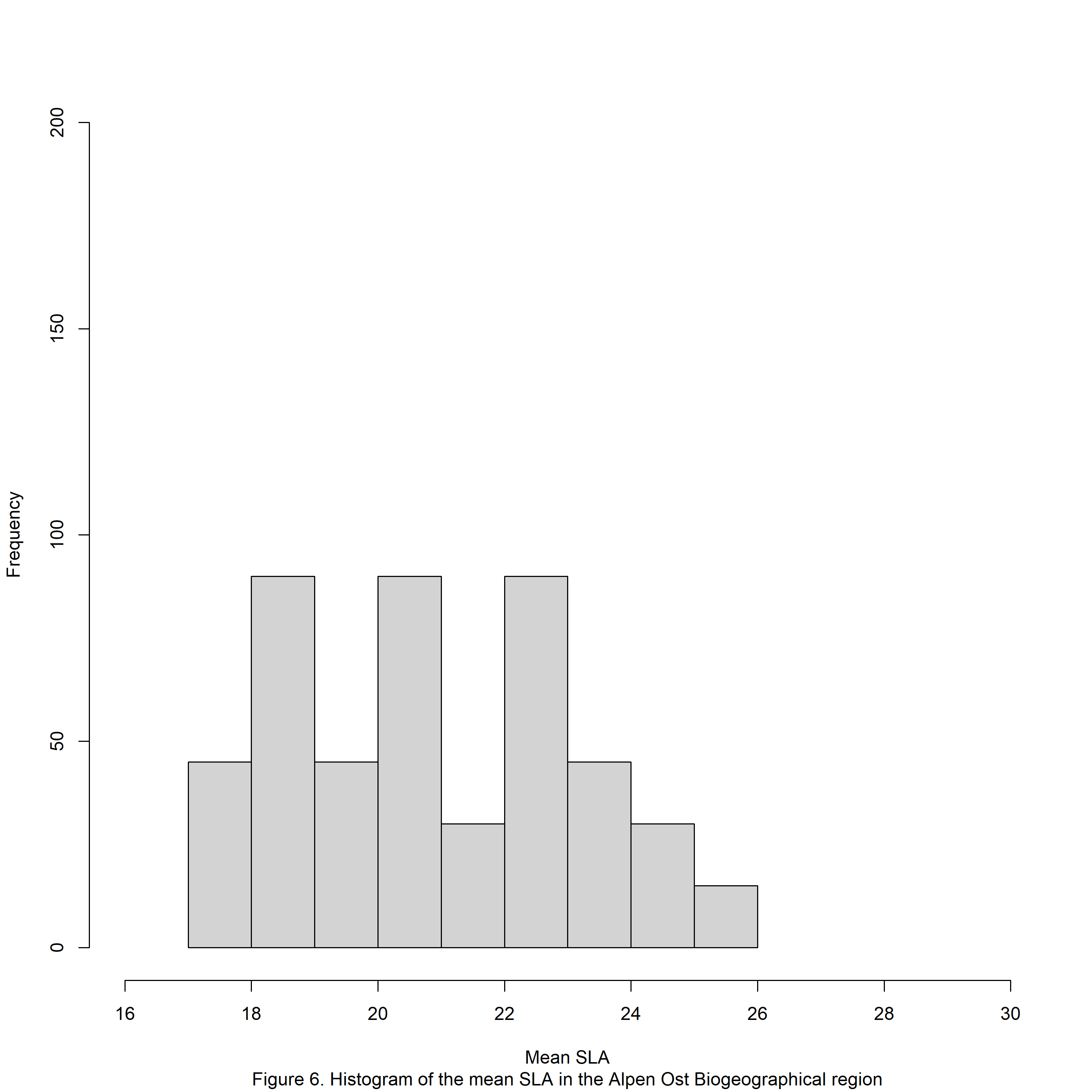

Supplement: Supplementary file 10 — Appendix S5‐6 [file ECE3-10-9906-s010.tiff]

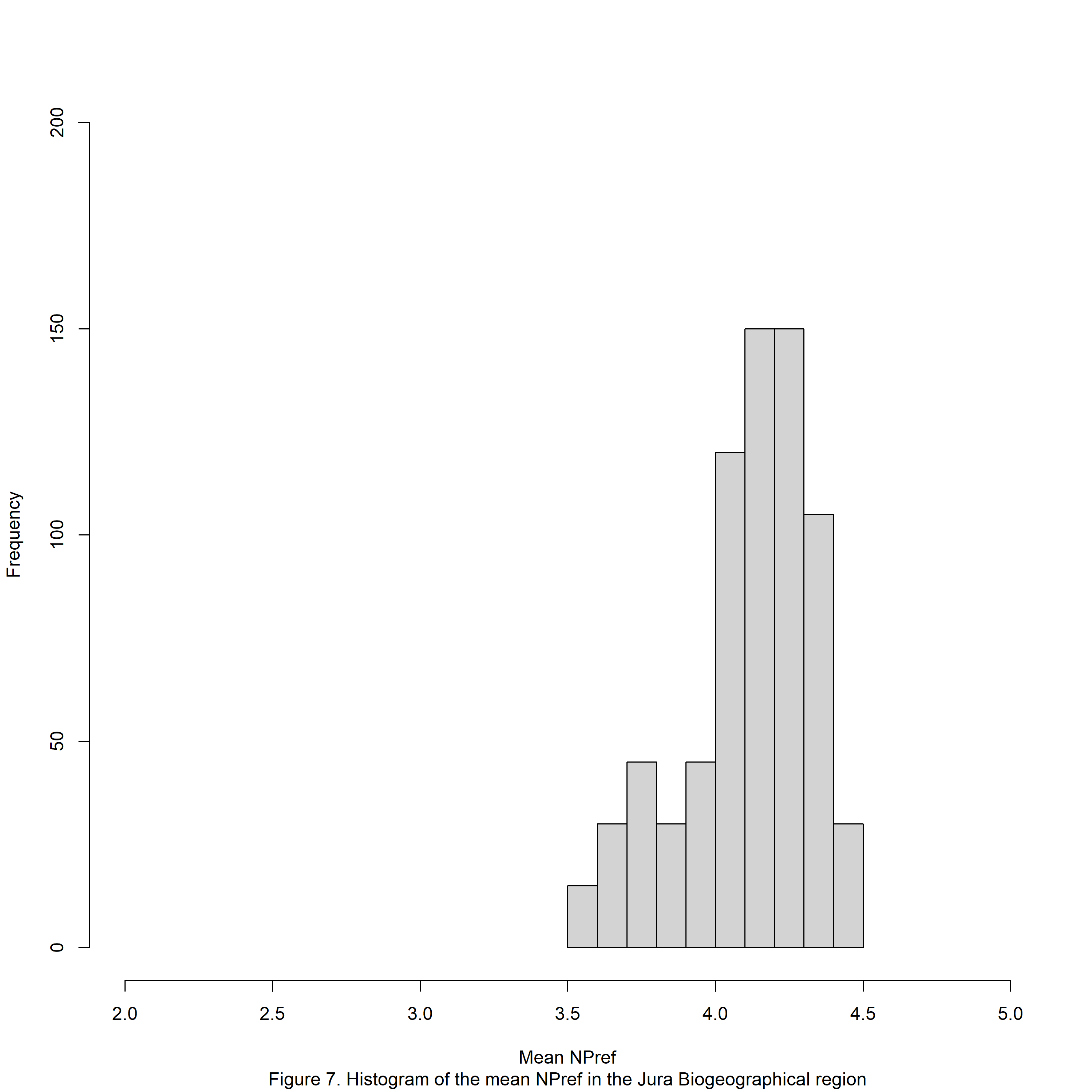

Supplement: Supplementary file 11 — Appendix S5‐7 [file ECE3-10-9906-s011.tiff]

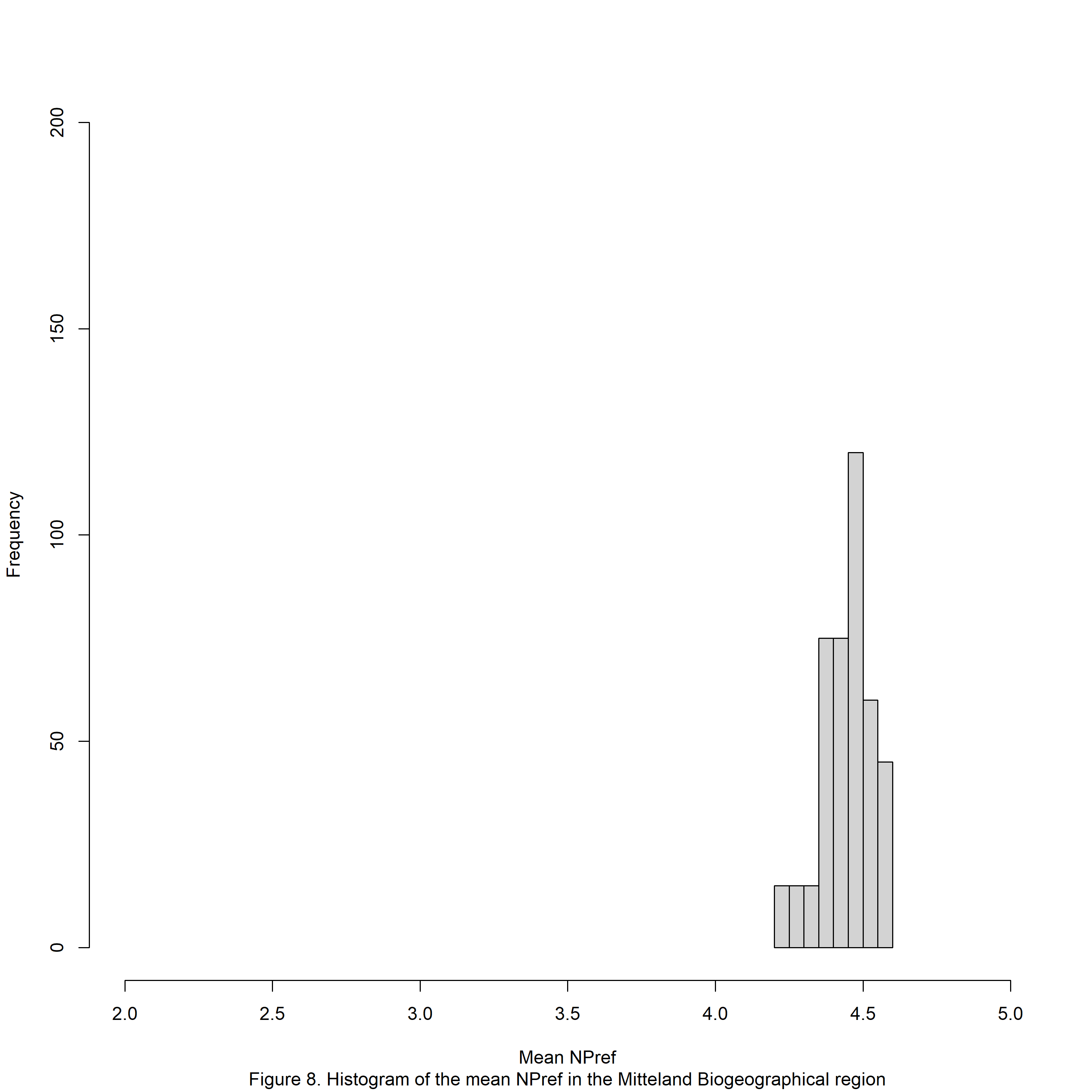

Supplement: Supplementary file 12 — Appendix S5‐8 [file ECE3-10-9906-s012.tiff]

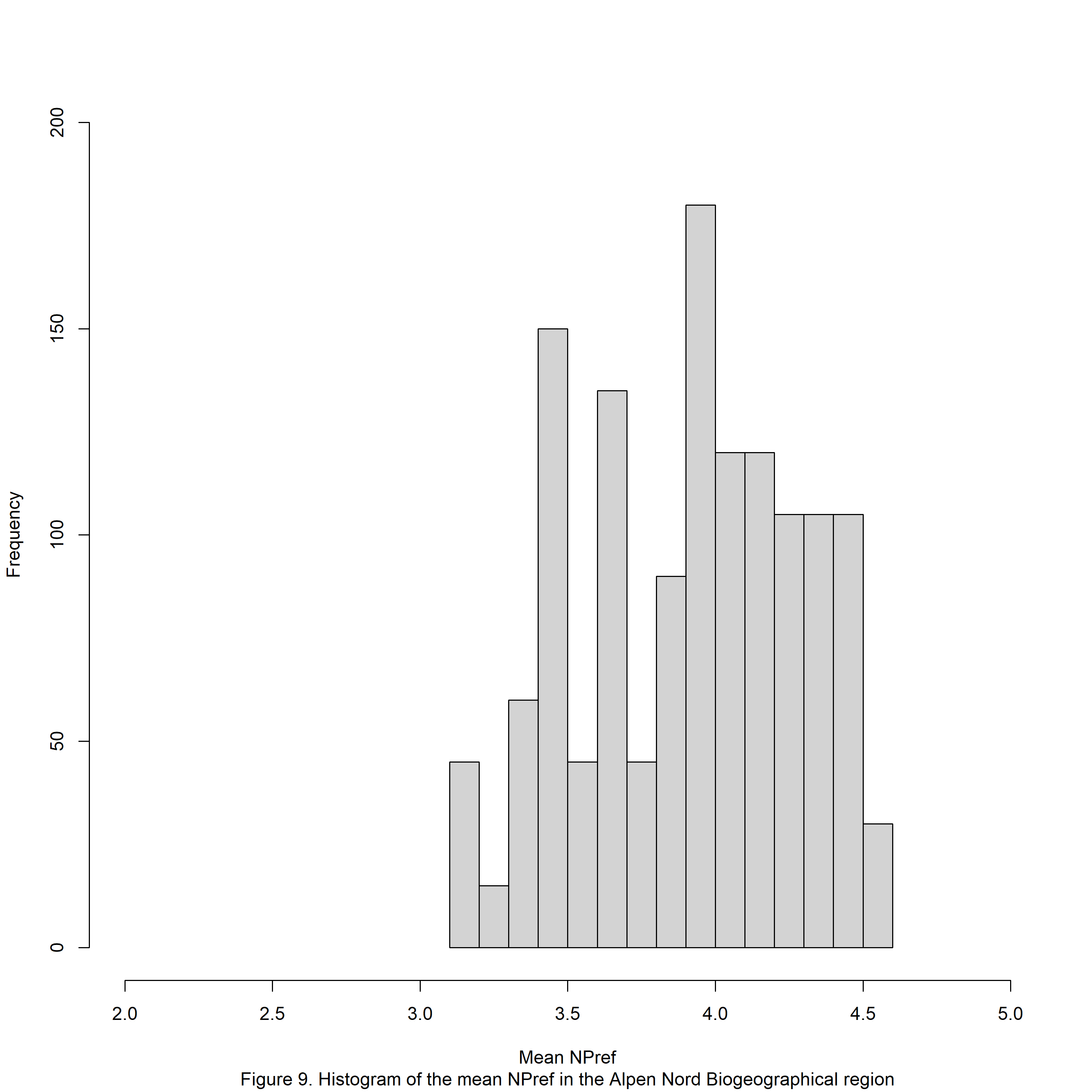

Supplement: Supplementary file 13 — Appendix S5‐9 [file ECE3-10-9906-s013.tiff]

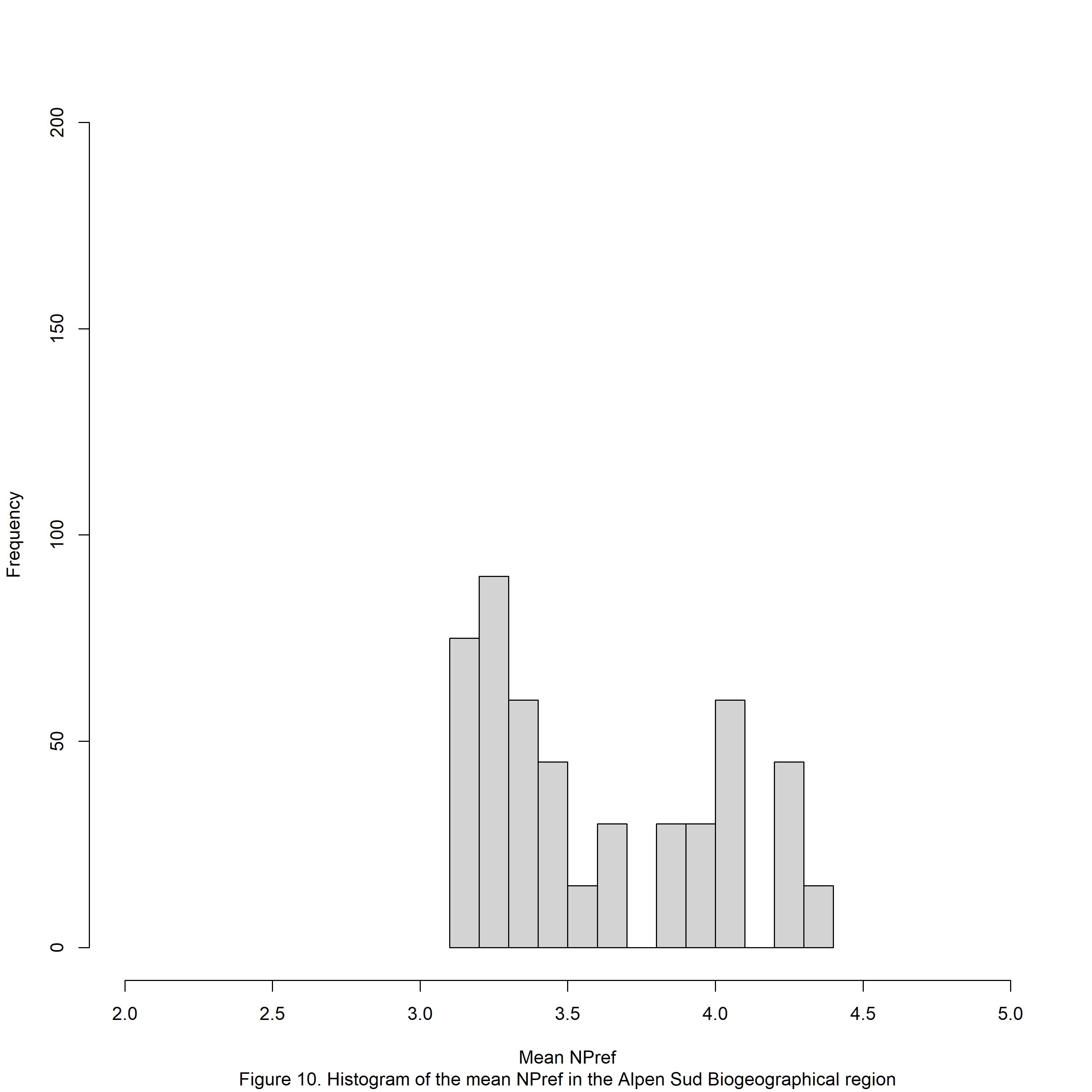

Supplement: Supplementary file 14 — Appendix S5‐10 [file ECE3-10-9906-s014.tiff]

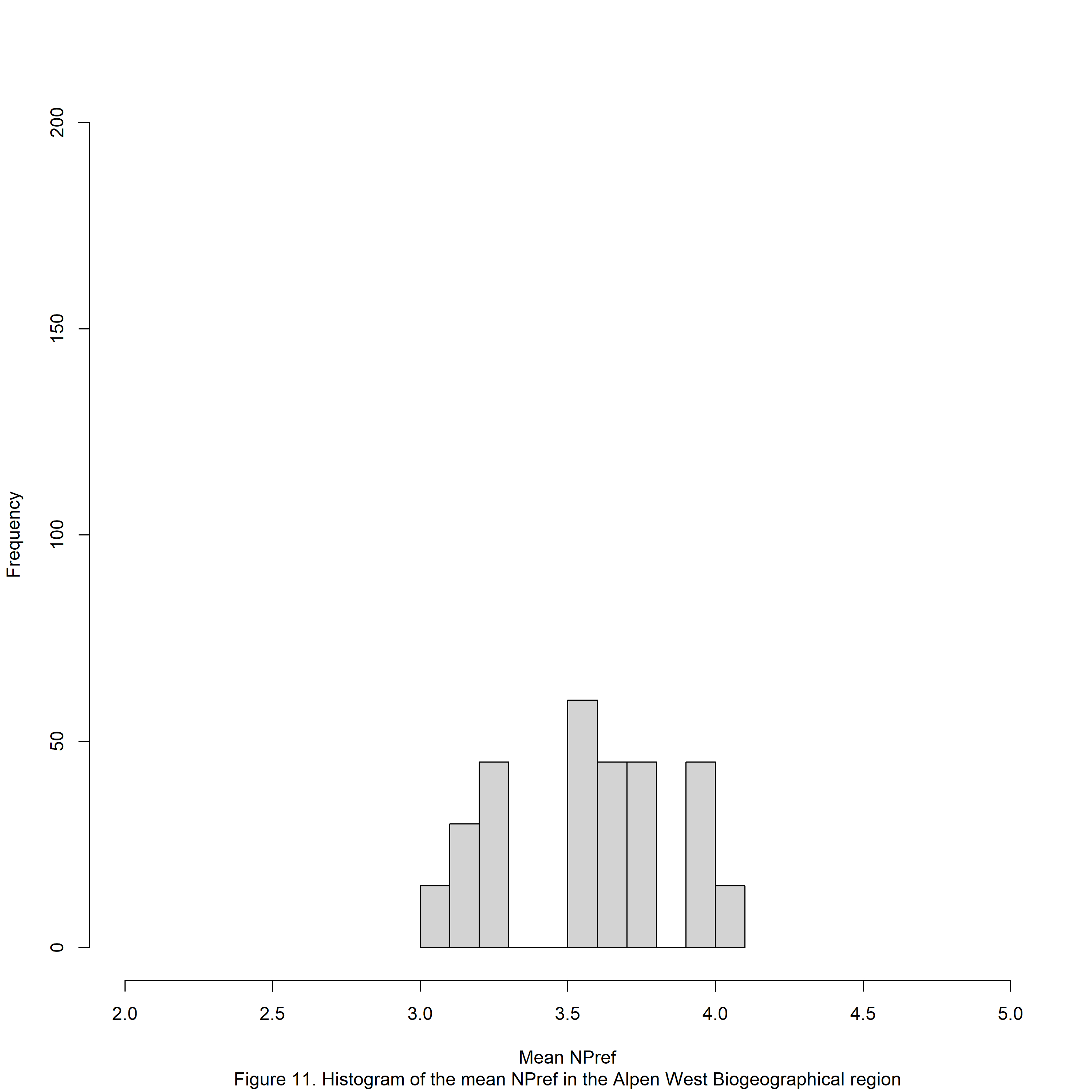

Supplement: Supplementary file 15 — Appendix S5‐11 [file ECE3-10-9906-s015.tiff]

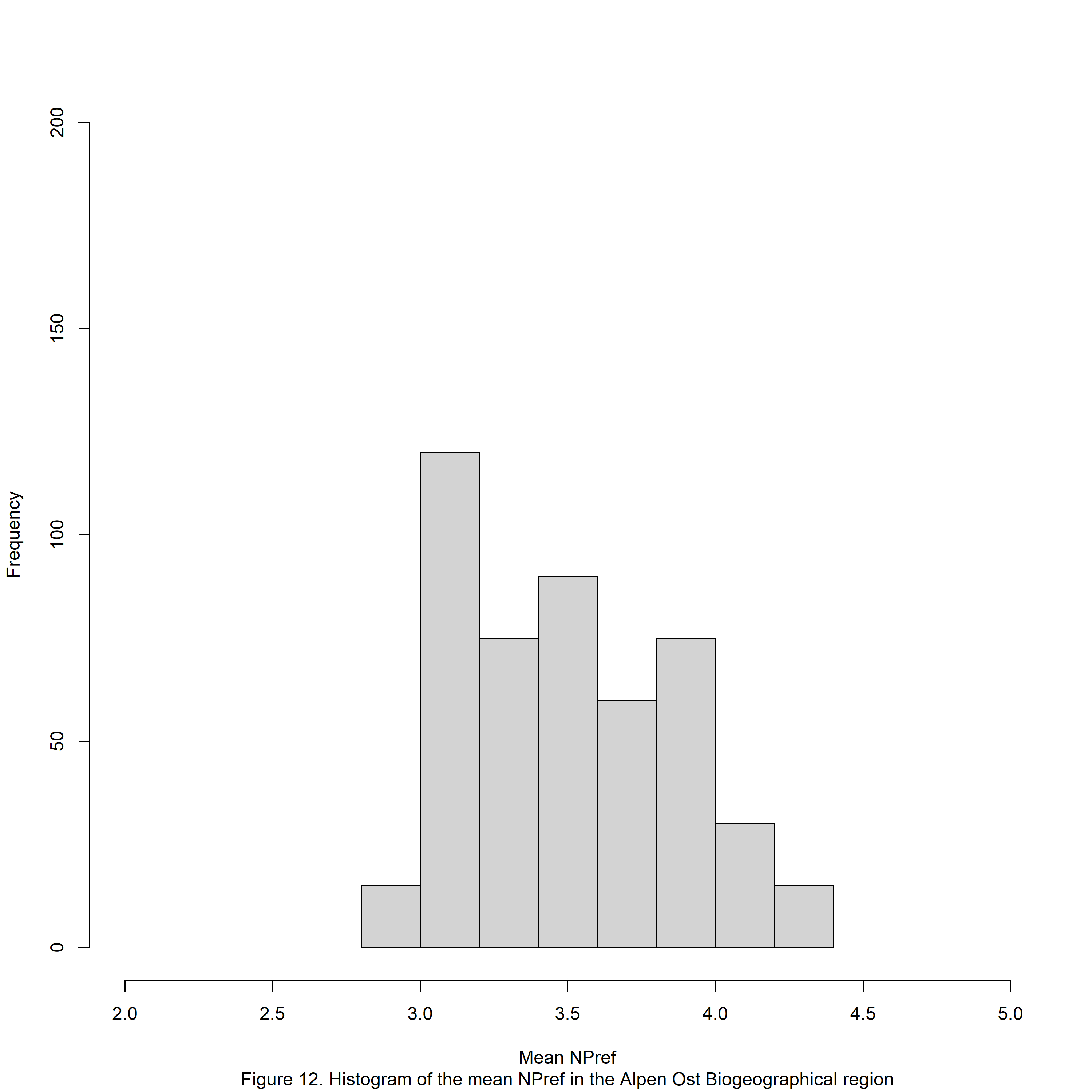

Supplement: Supplementary file 16 — Appendix S5‐12 [file ECE3-10-9906-s016.tiff]

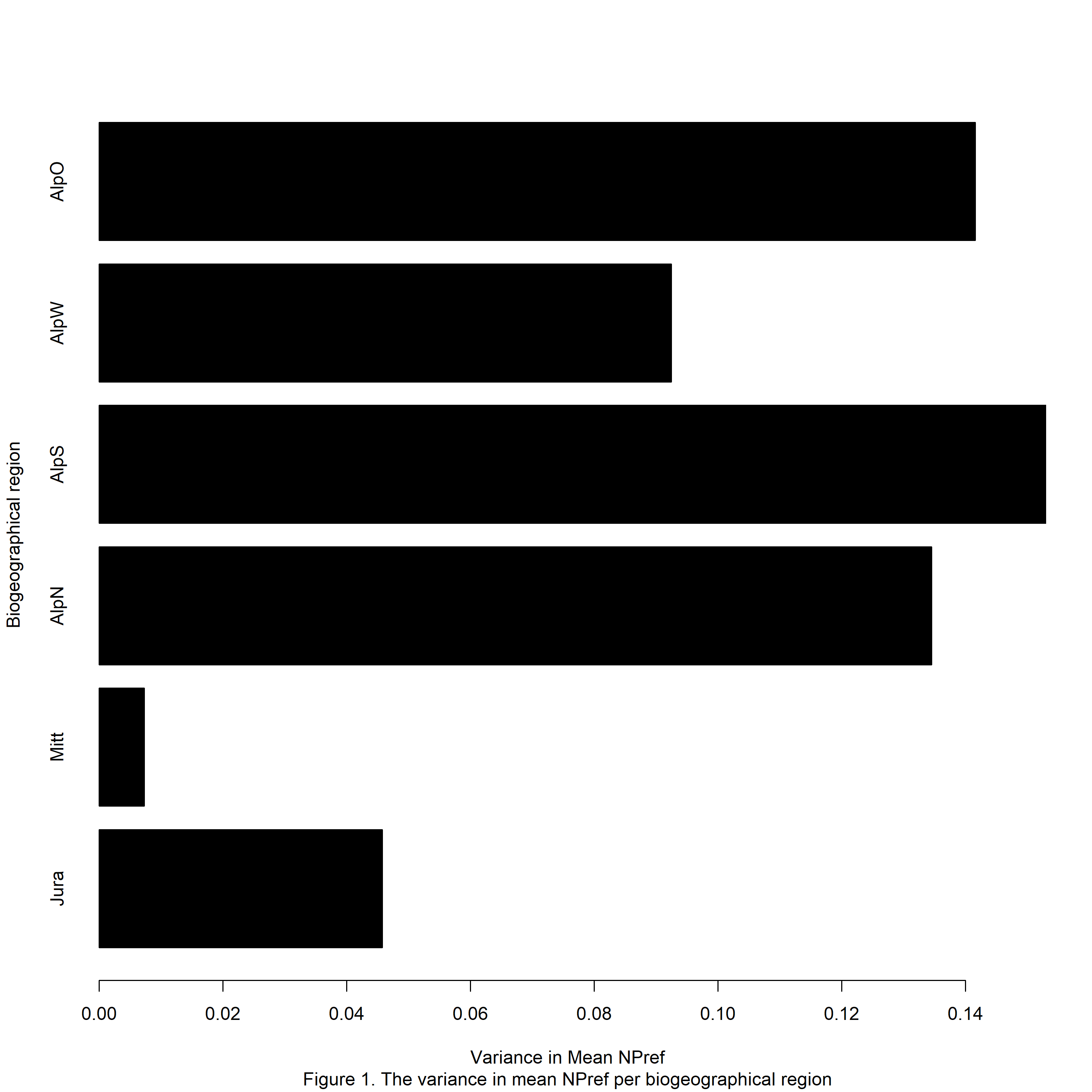

Supplement: Supplementary file 17 — Appendix S6‐1 [file ECE3-10-9906-s017.tiff]

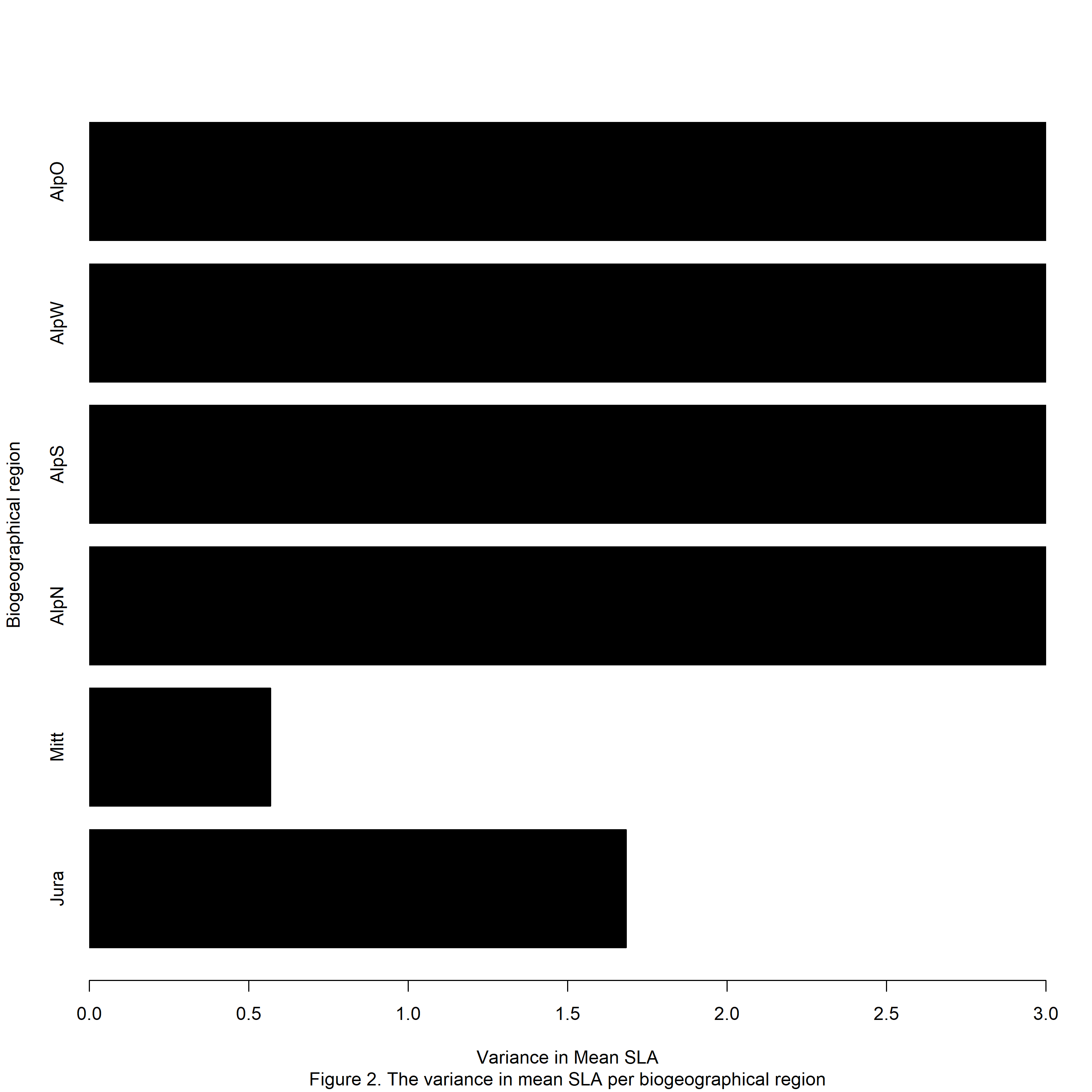

Supplement: Supplementary file 18 — Appendix S6‐2 [file ECE3-10-9906-s018.tiff]
