# Supplementary material for: A landscape‐scale assessment of the relationship between grassland functioning, community diversity, and functional traits
Source: Ecol Evol. 2020 Aug 16;10(18):9906–19. doi: 10.1002/ece3.6650 (PMC7520175; doi:10.1002/ece3.6650)

**Appendix I**

See Appendix III for an overview of the biogeographical regions of Switzerland. Jura = Jura, Mitt = Mittelland, AlpO = Östliche Zentralalpen, AlpN = Alpennordflanke, AlpS = Alpensüdflanke, and AlpW = Westliche Zentralalpen.


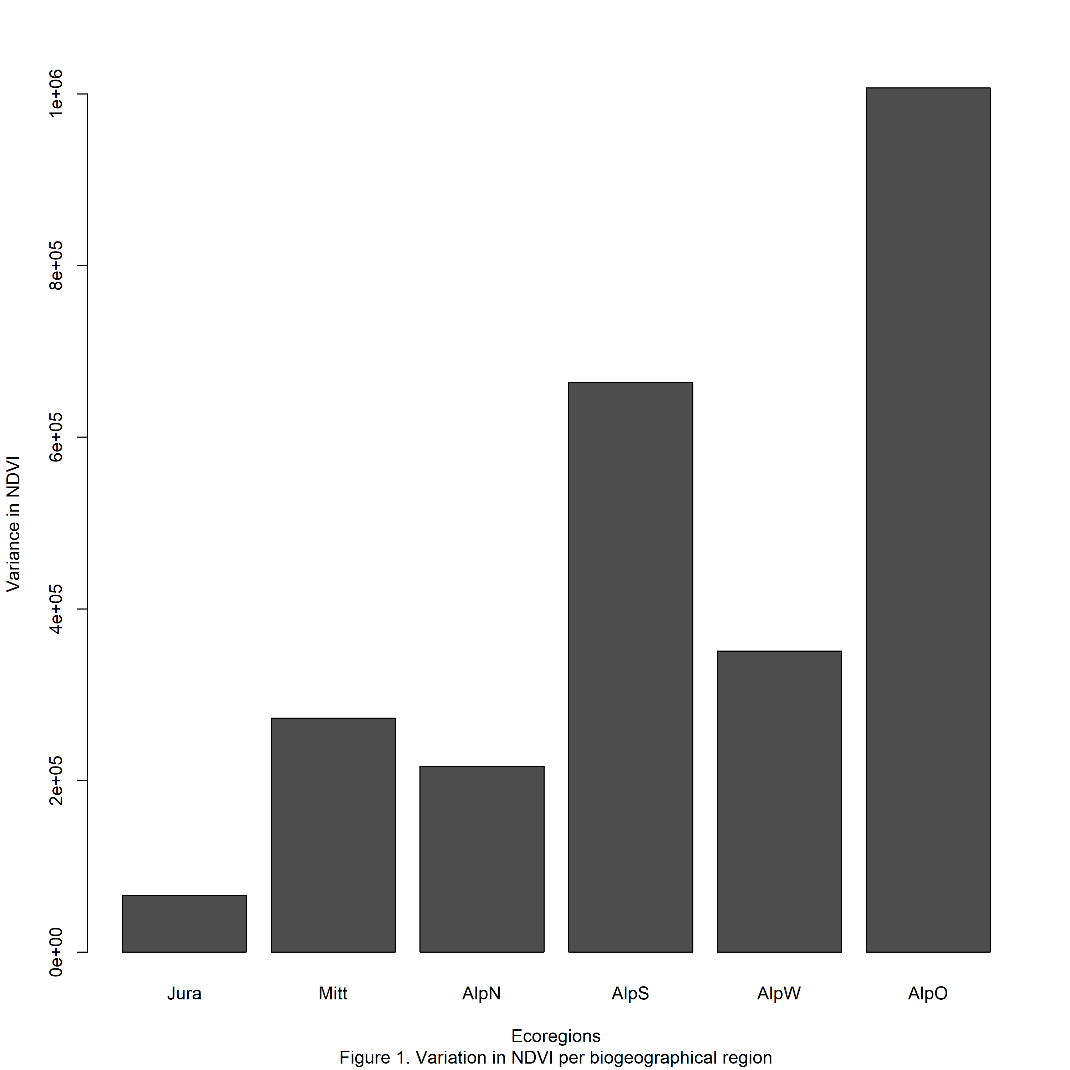


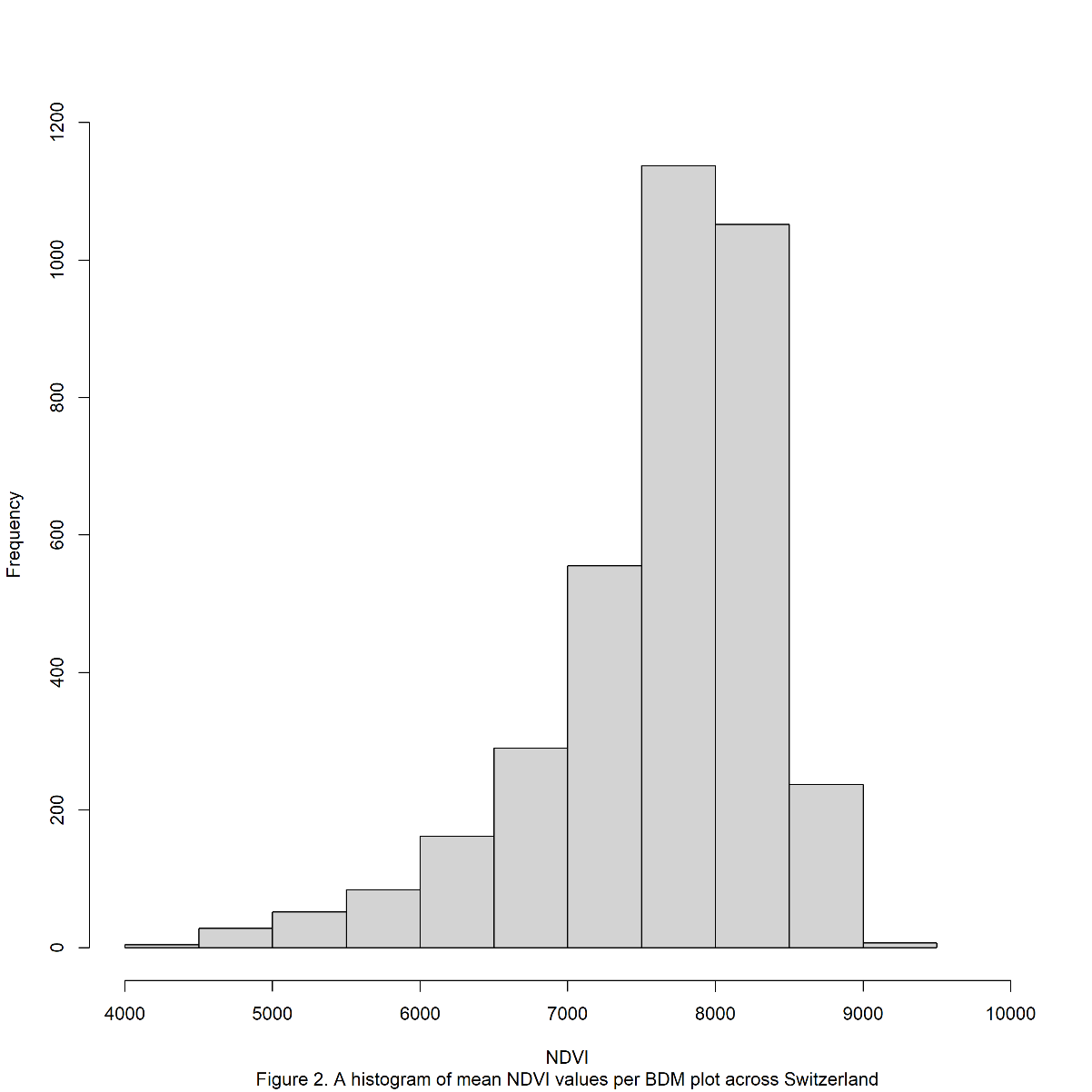


**Appendix II**

This table includes the twenty models that best explain grassland productivity (NDVI). The higher the AICc value, the better the model predicts productivity. A + sign or a number signals that the variable is included in the model. The + sign is shown for those variables that are quadrated in model (Feve NPref, Fdiv NPref, Mean NPref, Mean SLA and Temperature). We quadrated these variables because we observed a quadratic relationship with productivity for each of them. Fdiv = functional diversity, Feve = functional evenness.

| **Intercept** | **Feve SLA** | **Fdiv SLA** | **Feve NPref** | **Fdive NPref** | **Mean NPref** | **Mean SLA** | **Temperature** | **Precipitation** | **Richness** | **df** | **logLik** | **AICc** | **delta** | **weight** |
| --- | --- | --- | --- | --- | --- | --- | --- | --- | --- | --- | --- | --- | --- | --- |
| -7.77E-05 | NA | NA | + | + | + | + | + | NA | NA | 14 | -2126.49 | 4281.105 | 0 | 0.559143 |
| -0.0009254 | NA | NA | + | + | + | + | + | 0.038045892 | NA | 15 | -2126.48 | 4283.084 | 1.978861 | 0.207883 |
| 0.00024269 | NA | 0.060802635 | + | + | + | + | + | NA | NA | 15 | -2127.68 | 4285.489 | 4.383694 | 0.062462 |
| -0.0002942 | NA | NA | + | + | + | + | + | NA | -0.0143089 | 15 | -2128.33 | 4286.801 | 5.695342 | 0.032419 |
| -0.0001932 | 0.029456289 | NA | + | + | + | + | + | NA | NA | 15 | -2128.48 | 4287.088 | 5.982434 | 0.028084 |
| -0.0006058 | NA | 0.059566888 | + | + | + | + | + | 0.037790883 | NA | 16 | -2127.7 | 4287.556 | 6.450375 | 0.022225 |
| -8.69E-05 | NA | NA | + | NA | + | + | + | NA | NA | 12 | -2131.8 | 4287.679 | 6.573783 | 0.020895 |
| -0.0011672 | NA | NA | + | + | + | + | + | 0.038084658 | -0.0159155 | 16 | -2128.31 | 4288.769 | 7.663421 | 0.012118 |
| -0.0010231 | 0.026286704 | NA | + | + | + | + | + | 0.037796224 | NA | 16 | -2128.5 | 4289.16 | 8.054452 | 0.009966 |
| -0.0009378 | NA | NA | + | NA | + | + | + | 0.038173098 | NA | 13 | -2131.75 | 4289.606 | 8.501071 | 0.007971 |
| 0.00011631 | 0.066273773 | 0.086058628 | + | + | + | + | + | NA | NA | 16 | -2128.84 | 4289.824 | 8.71874 | 0.007149 |
| -0.0001416 | NA | NA | + | + | + | NA | + | NA | NA | 12 | -2133 | 4290.086 | 8.980308 | 0.006273 |
| -0.0002726 | NA | 0.066064654 | + | + | + | + | + | NA | -0.03590927 | 16 | -2129.36 | 4290.872 | 9.766693 | 0.004234 |
| -0.0009932 | NA | NA | + | + | + | NA | + | 0.038323053 | NA | 13 | -2132.93 | 4291.963 | 10.85752 | 0.002454 |
| -0.0007085 | 0.061935593 | 0.083189855 | + | + | + | + | + | 0.037099401 | NA | 17 | -2128.98 | 4292.137 | 11.03198 | 0.002249 |
| 0.00022705 | NA | 0.059600493 | + | NA | + | + | + | NA | NA | 13 | -2133.02 | 4292.144 | 11.03809 | 0.002242 |
| -0.0002961 | 0.028556901 | NA | + | + | + | + | + | NA | -0.00701855 | 16 | -2130.32 | 4292.79 | 11.68435 | 0.001623 |
| -0.0011408 | NA | 0.065008818 | + | + | + | + | + | 0.037862863 | -0.0371584 | 17 | -2129.37 | 4292.917 | 11.81194 | 0.001523 |
| -0.0006394 | NA | NA | + | NA | + | + | + | NA | -0.03660855 | 13 | -2133.51 | 4293.131 | 12.02538 | 0.001369 |
| -0.0002263 | 0.035819344 | NA | + | NA | + | + | + | NA | NA | 13 | -2133.68 | 4293.454 | 12.34852 | 0.001164 |

**Appendix III**

These are the biogeographical regions of Switzerland. In this article we abbreviate the biogeographical region as Jura = Jura, Mitt = Mittelland, AlpO = Östliche Zentralalpen, AlpN = Alpennordflanke, AlpS = Alpensüdflanke, and AlpW = Westliche Zentralalpen.


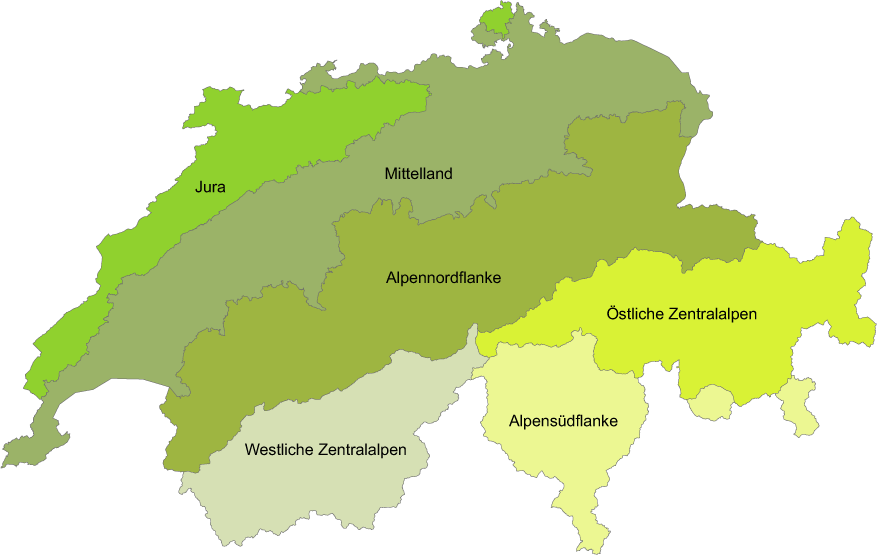


**Appendix IV**

The percentage of grassland species occurring in the BDM data that are included in the TRY database per biogeographical region (see Appendix III for an overview of the biogeographical regions of Switzerland). Jura = Jura, Mitt = Mittelland, AlpO = Östliche Zentralalpen, AlpN = Alpennordflanke, AlpS = Alpensüdflanke, and AlpW = Westliche Zentralalpen.

**
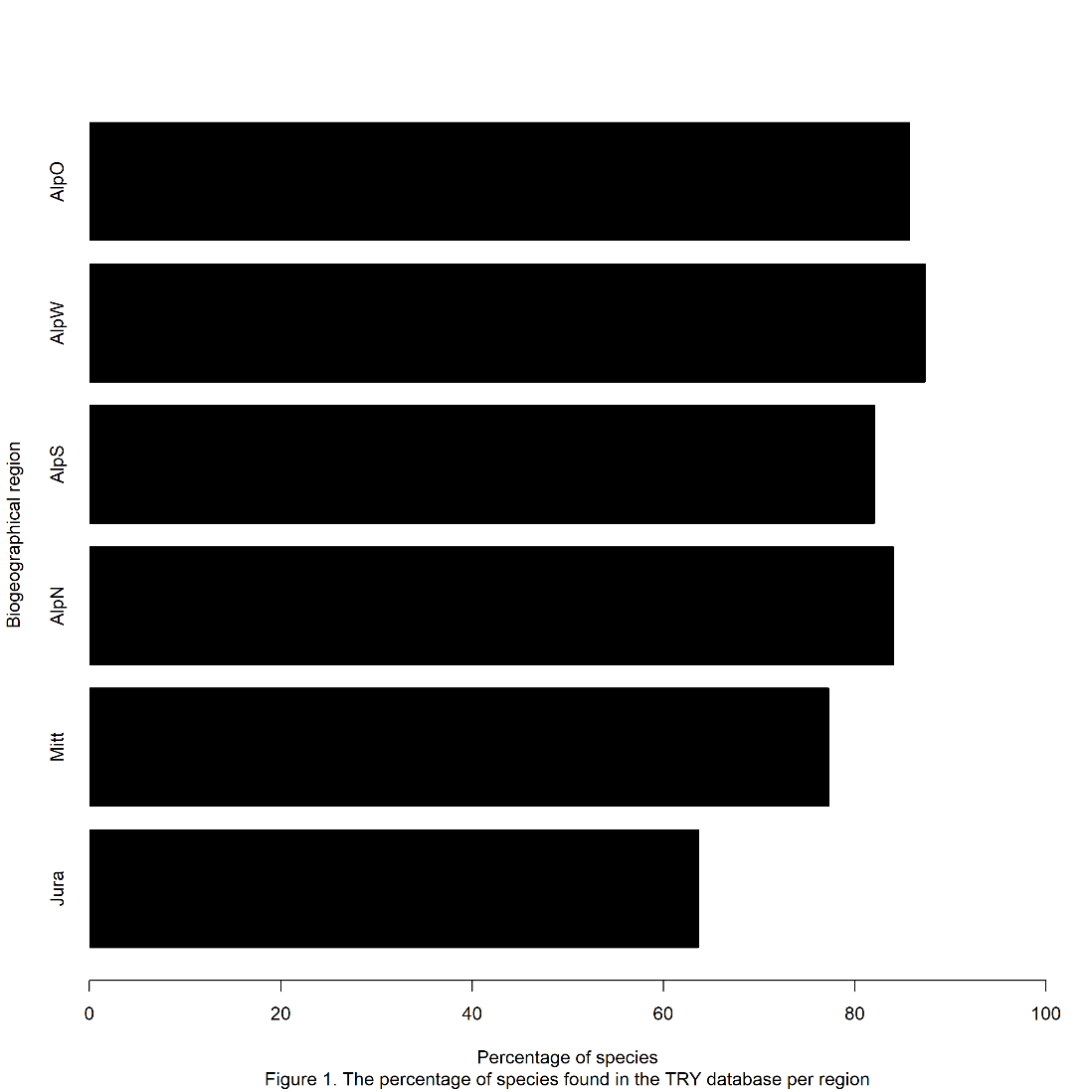
**

**Appendix V**

The distribution of mean SLA (specific leaf area) and mean NPref (nutrient preference) per biogeographical region of Switzerland (see Appendix III, Figure 1 for an overview of the biogeographical regions of Switzerland). Jura = Jura, Mitt = Mittelland, AlpO = Östliche Zentralalpen, AlpN = Alpennordflanke, AlpS = Alpensüdflanke, and AlpW = Westliche Zentralalpen.


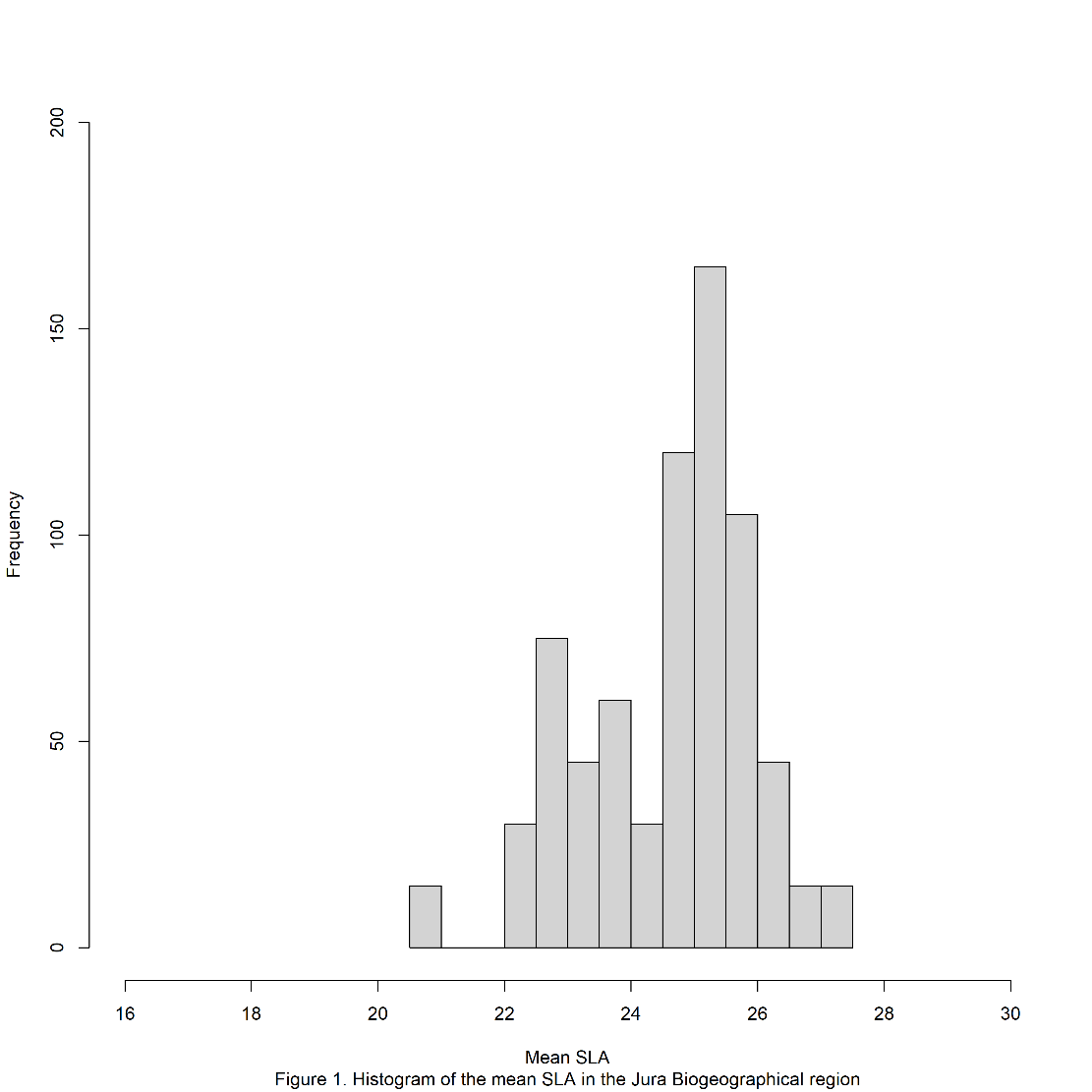

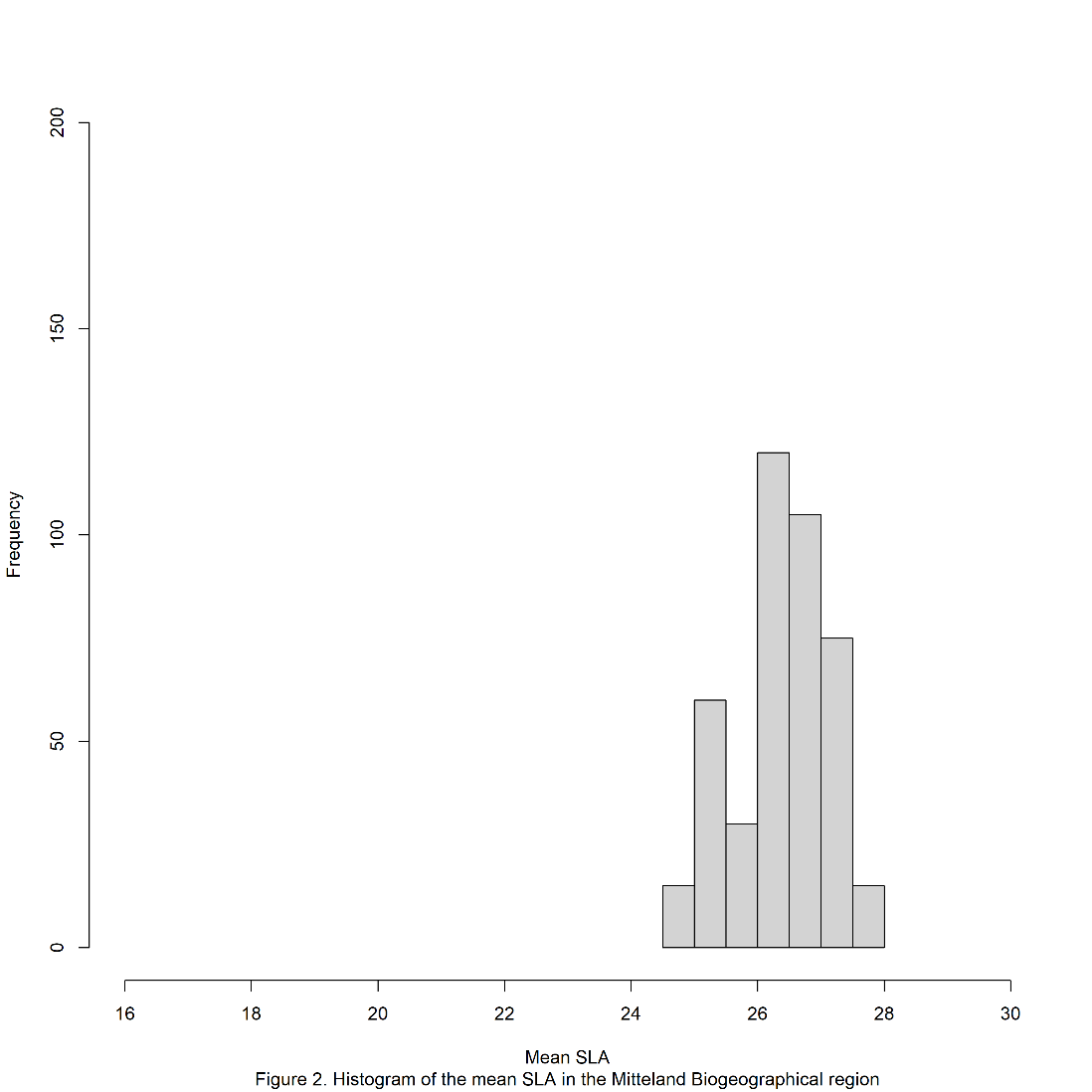


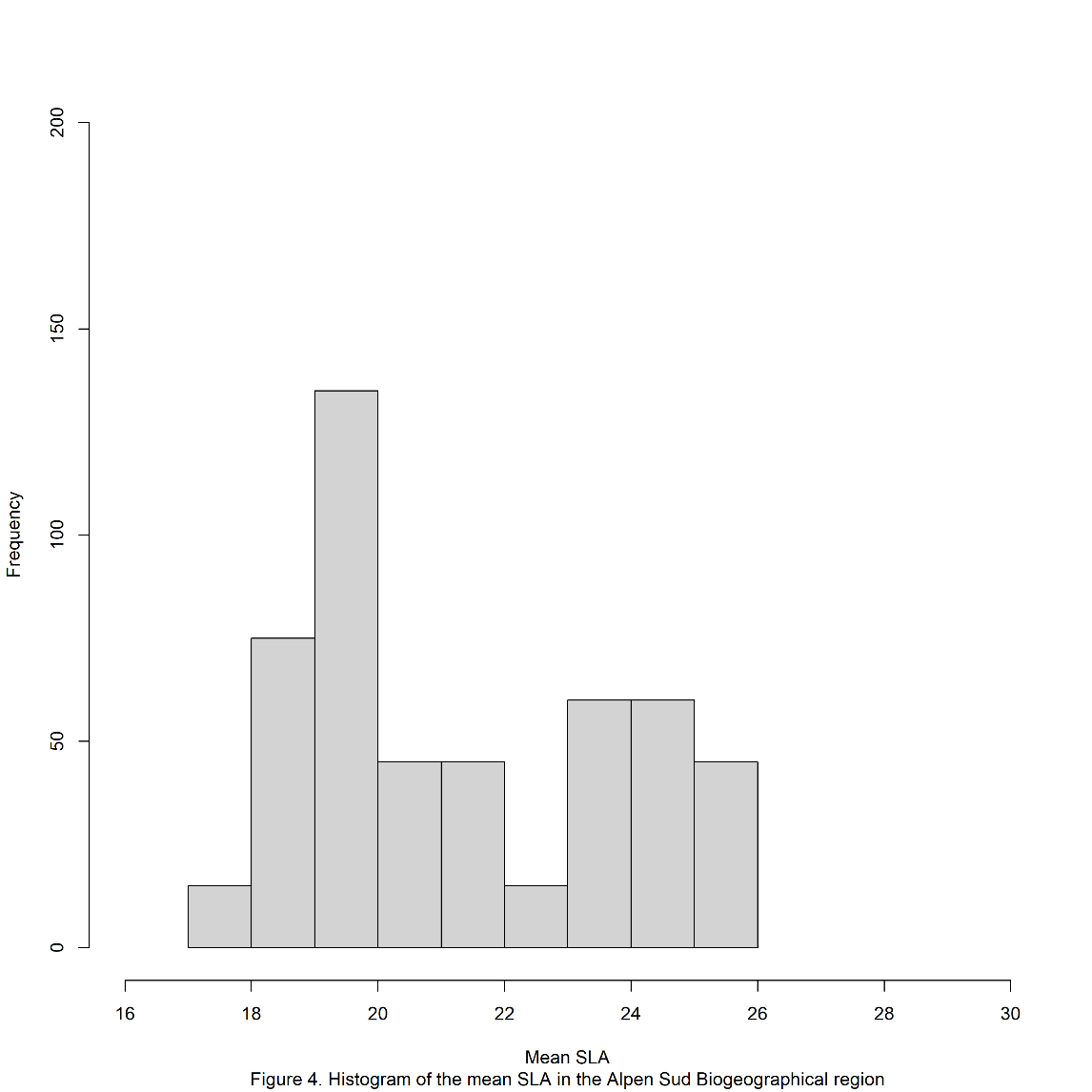

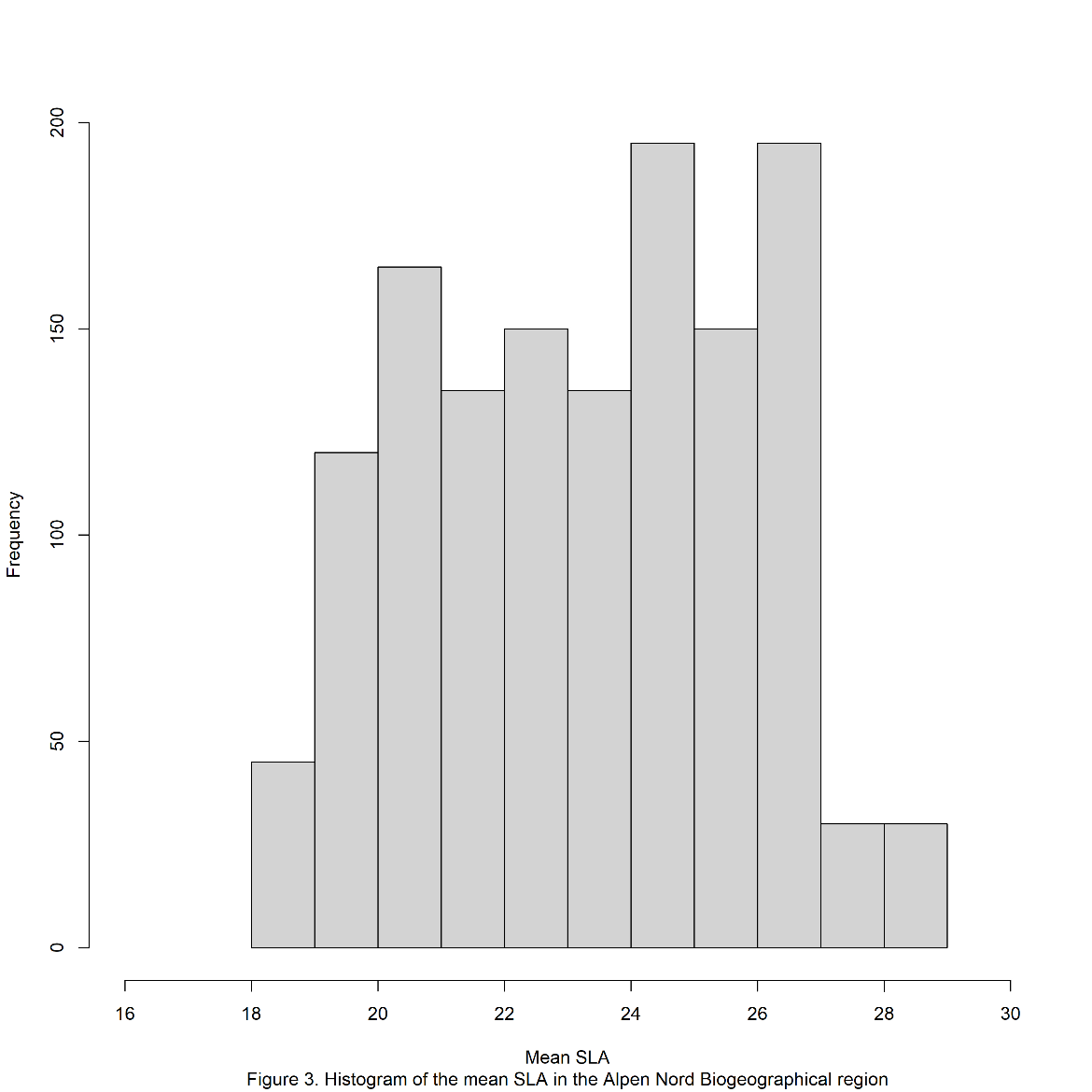


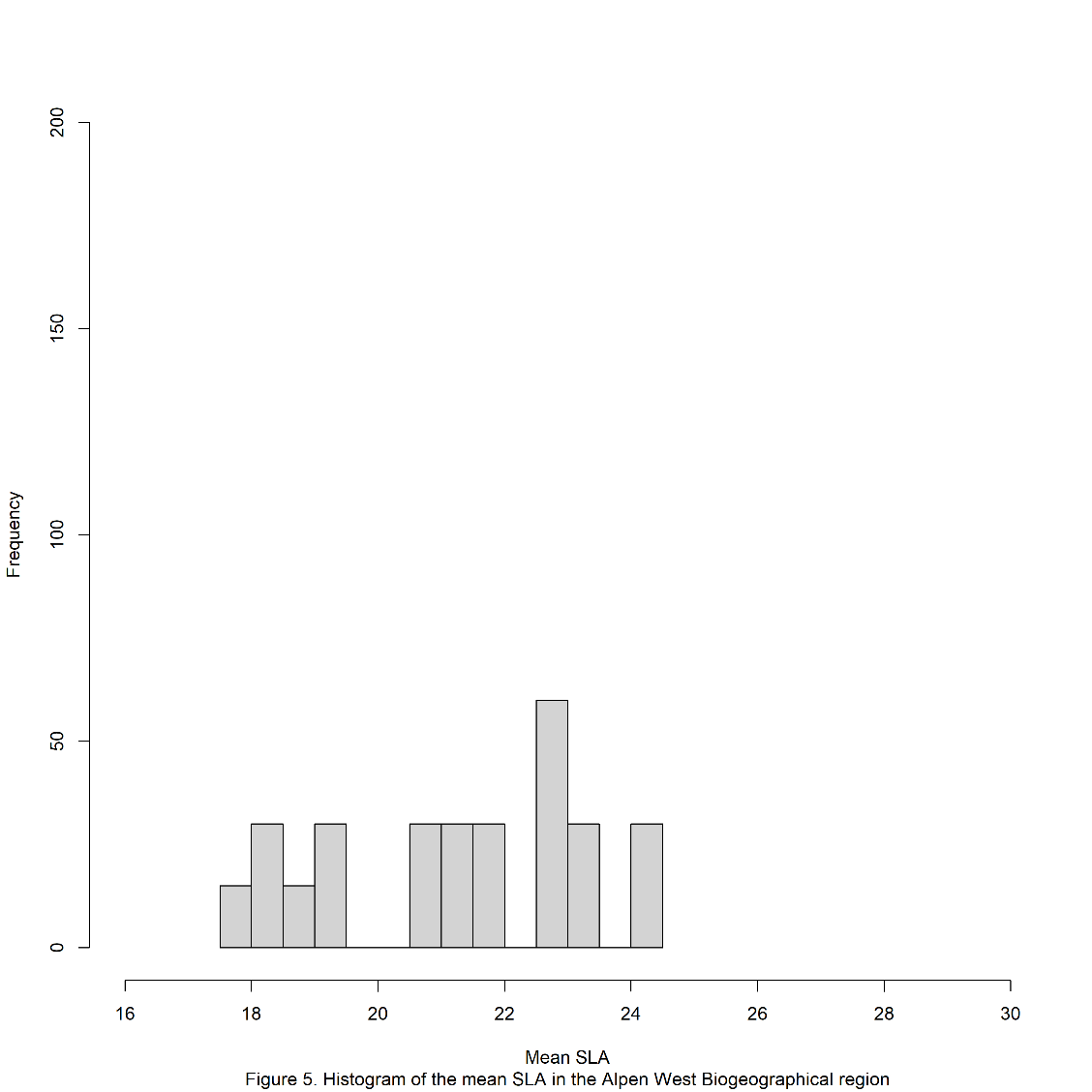

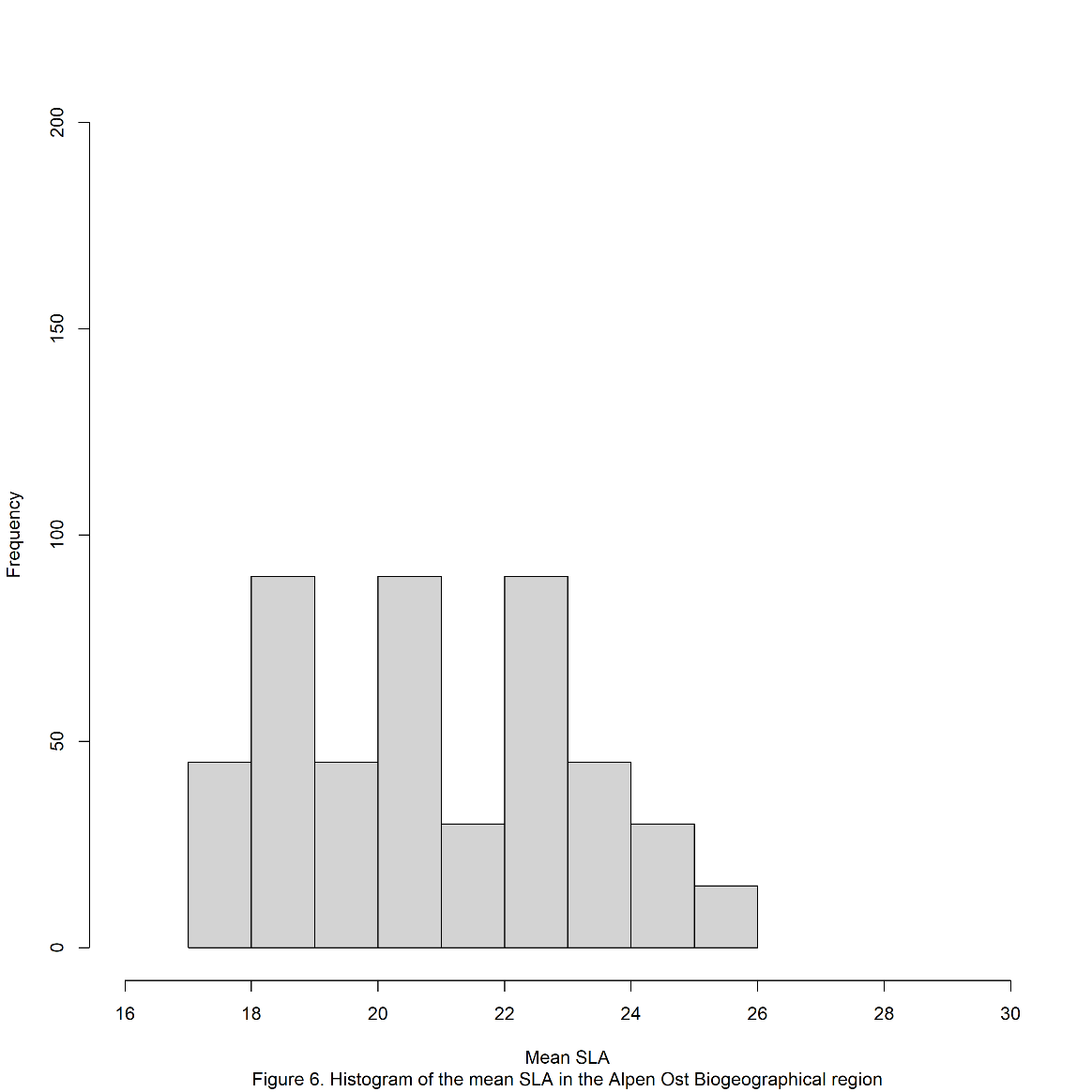


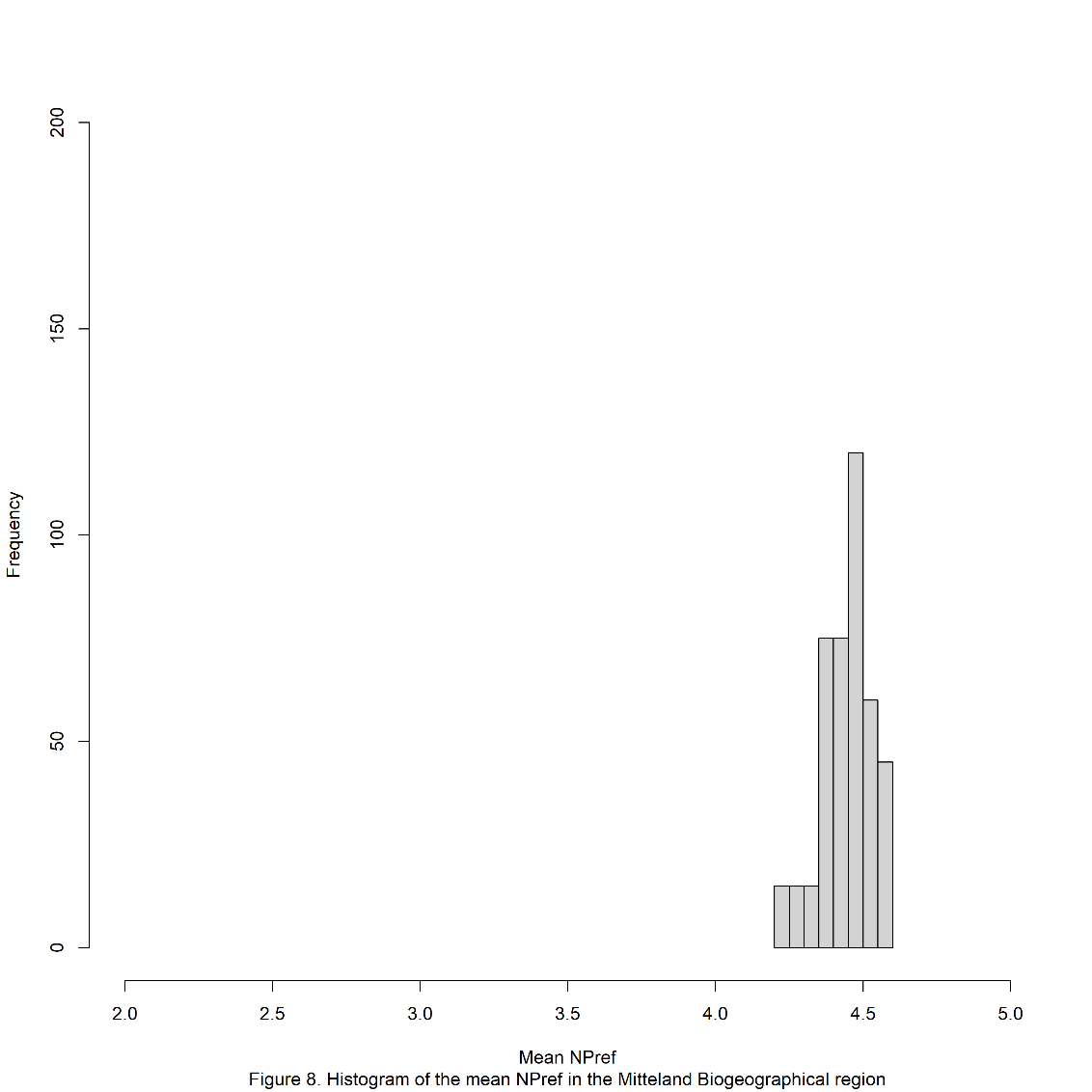


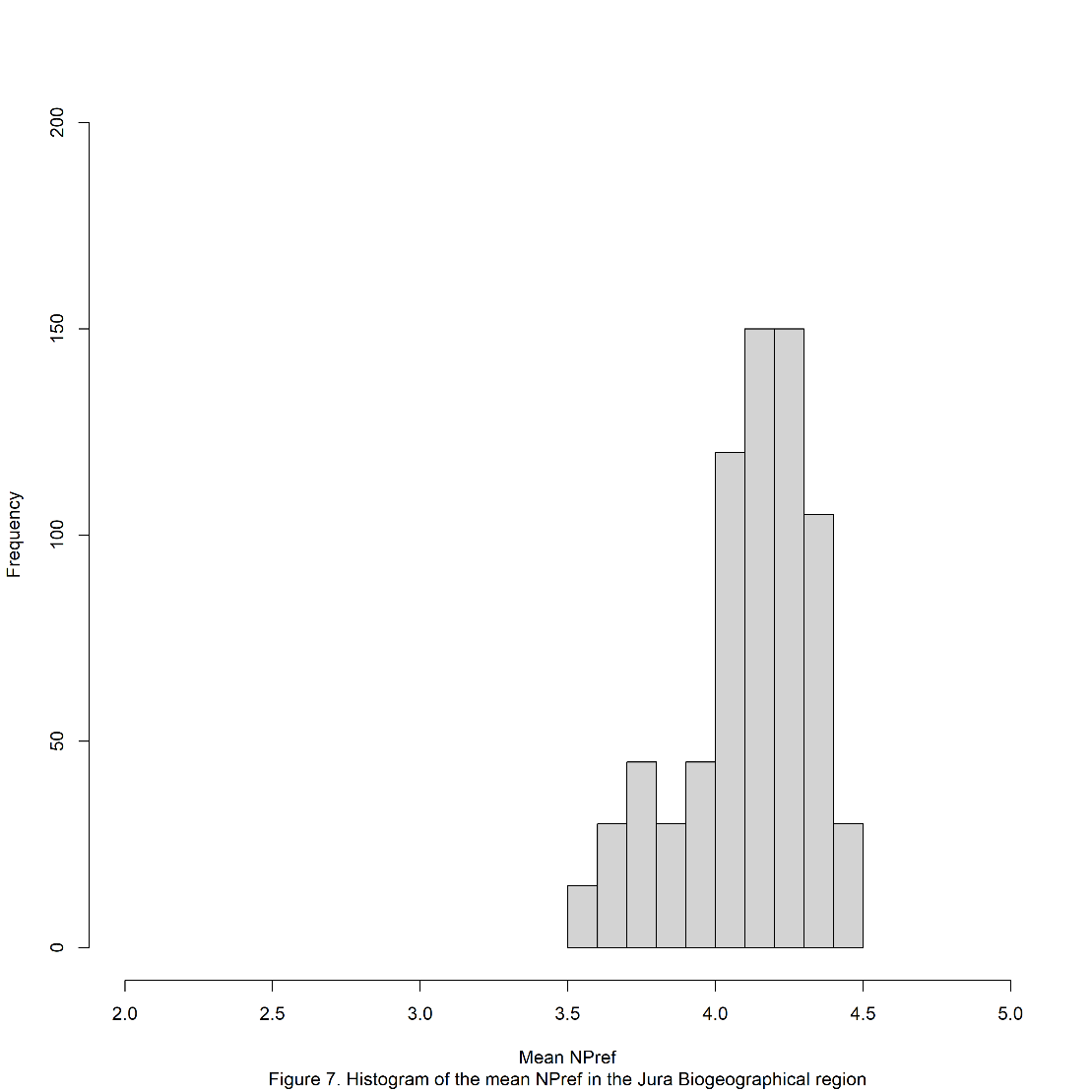


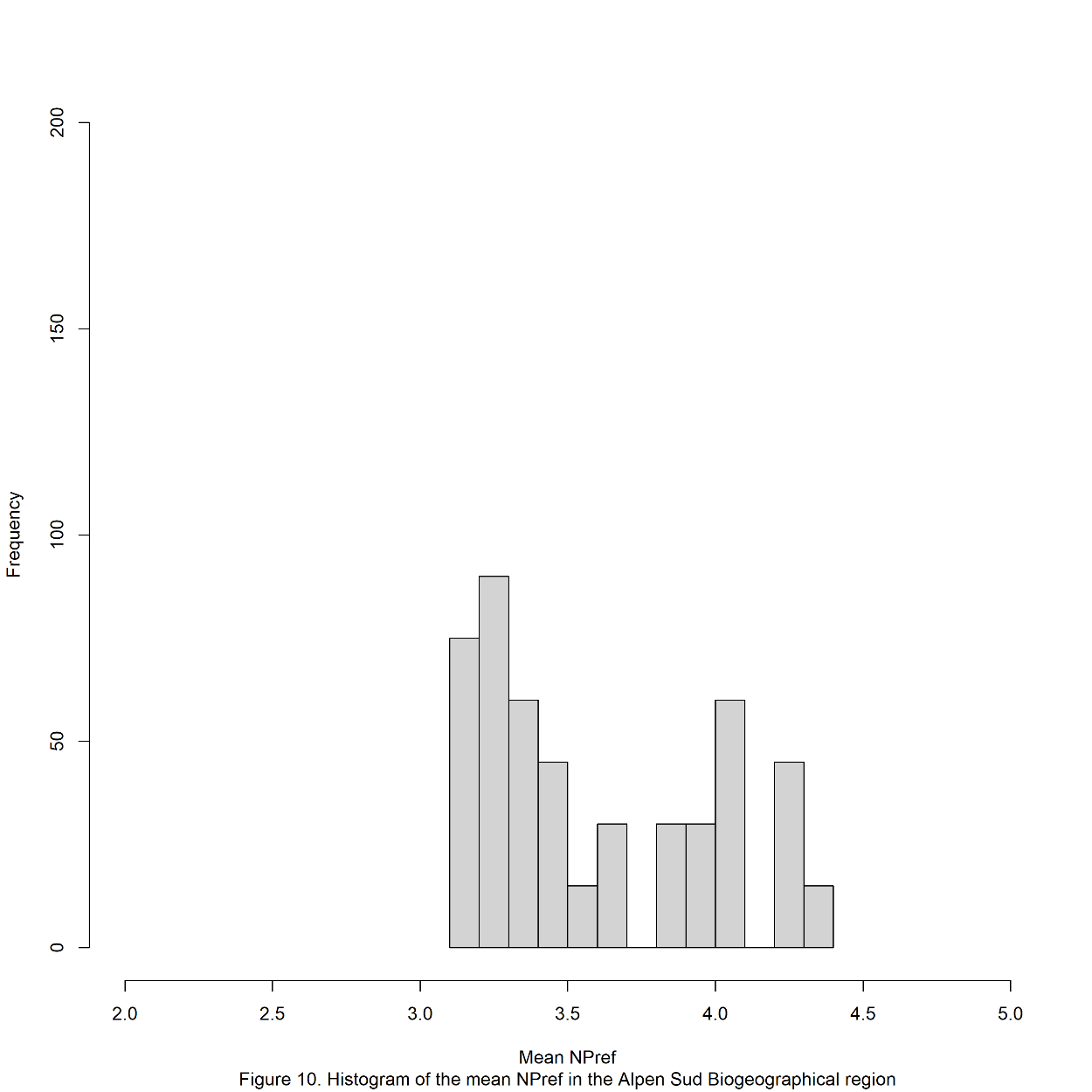


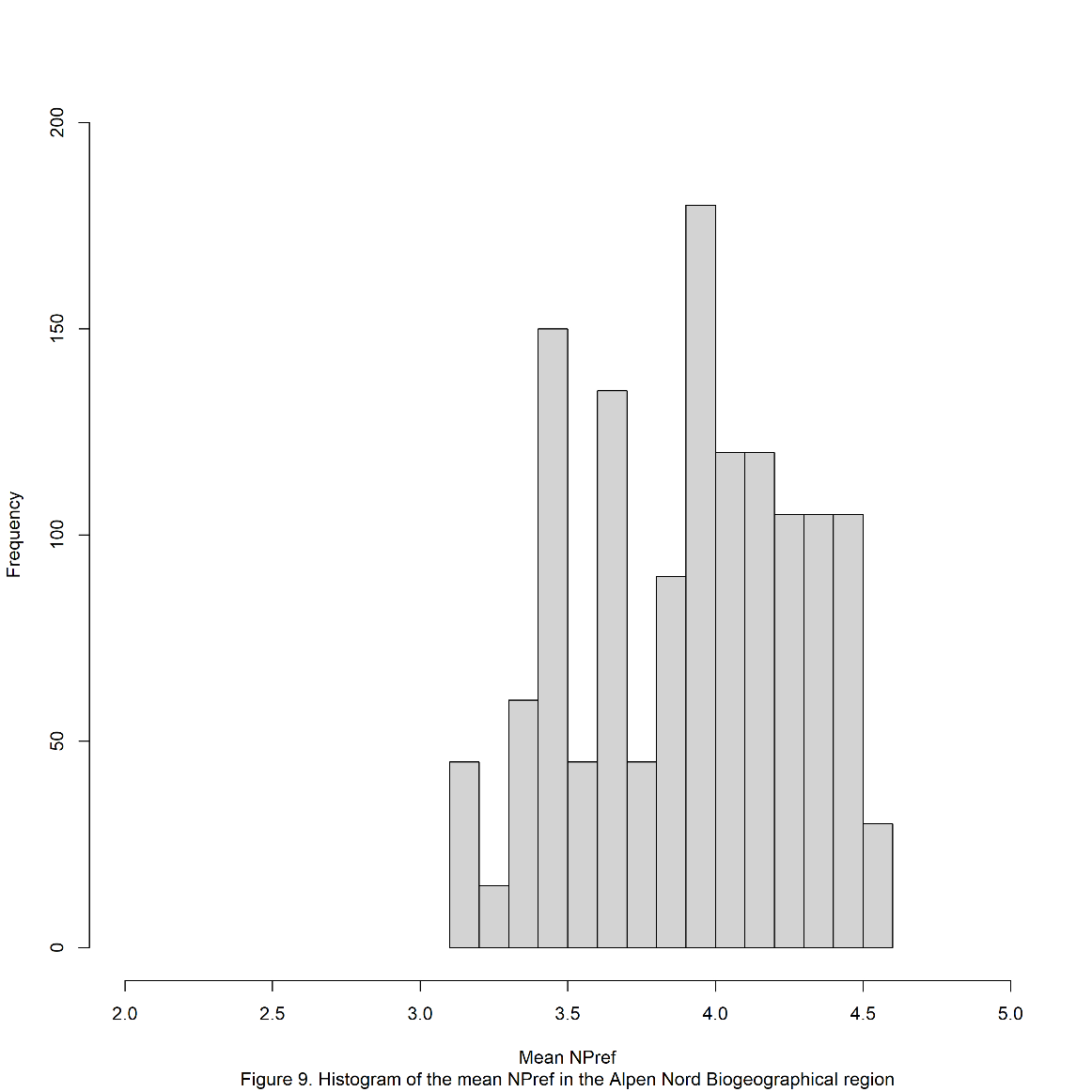


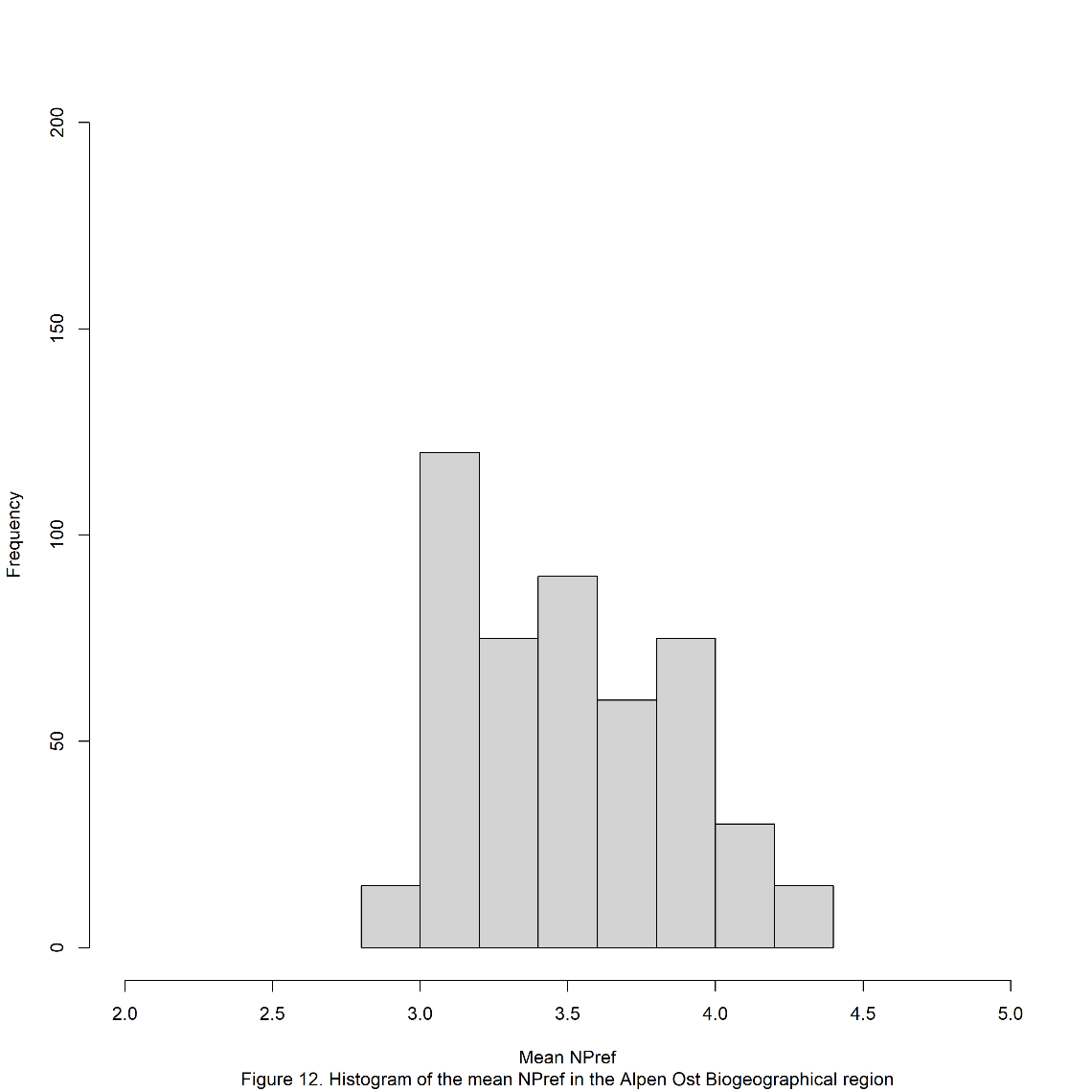

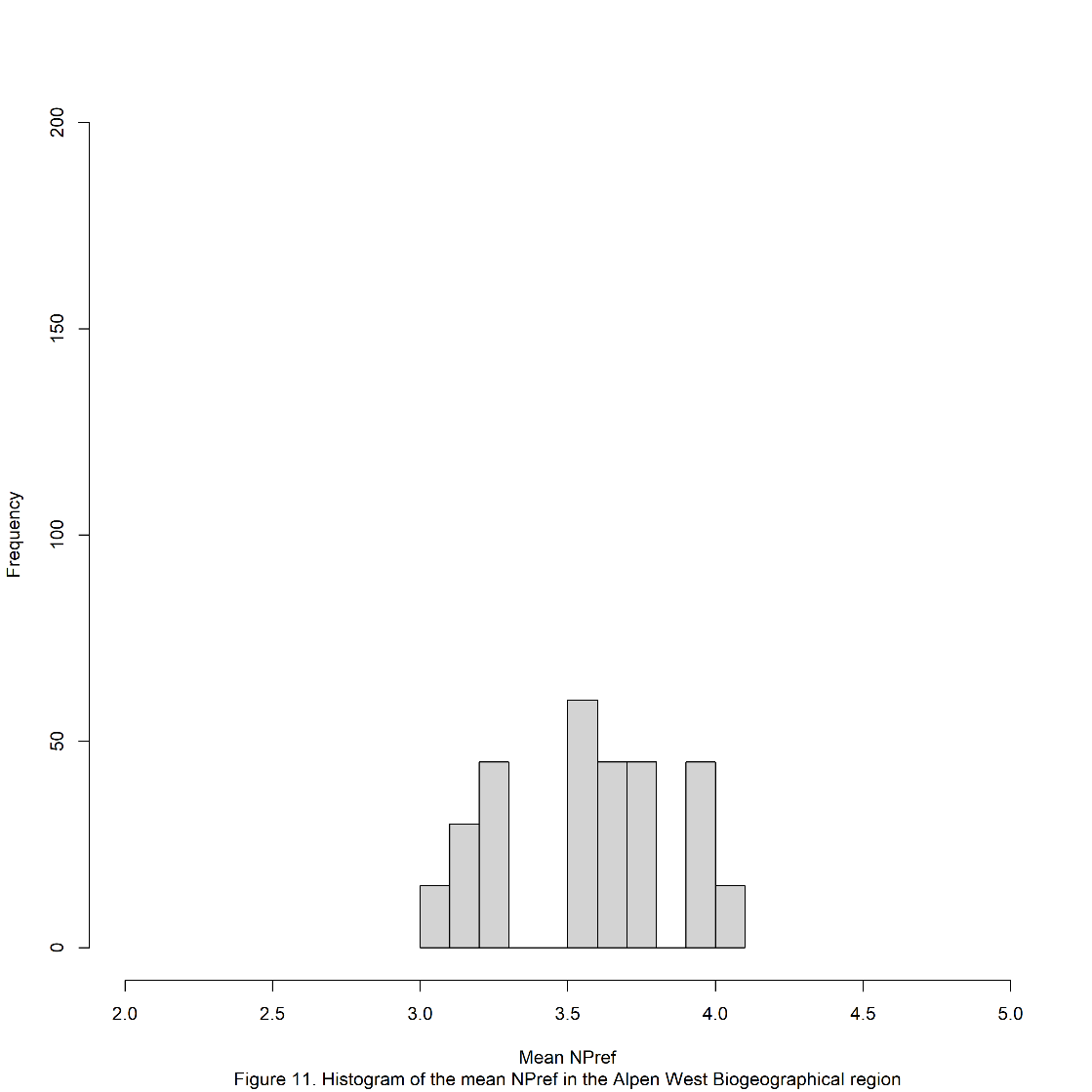


**Appendix VI**


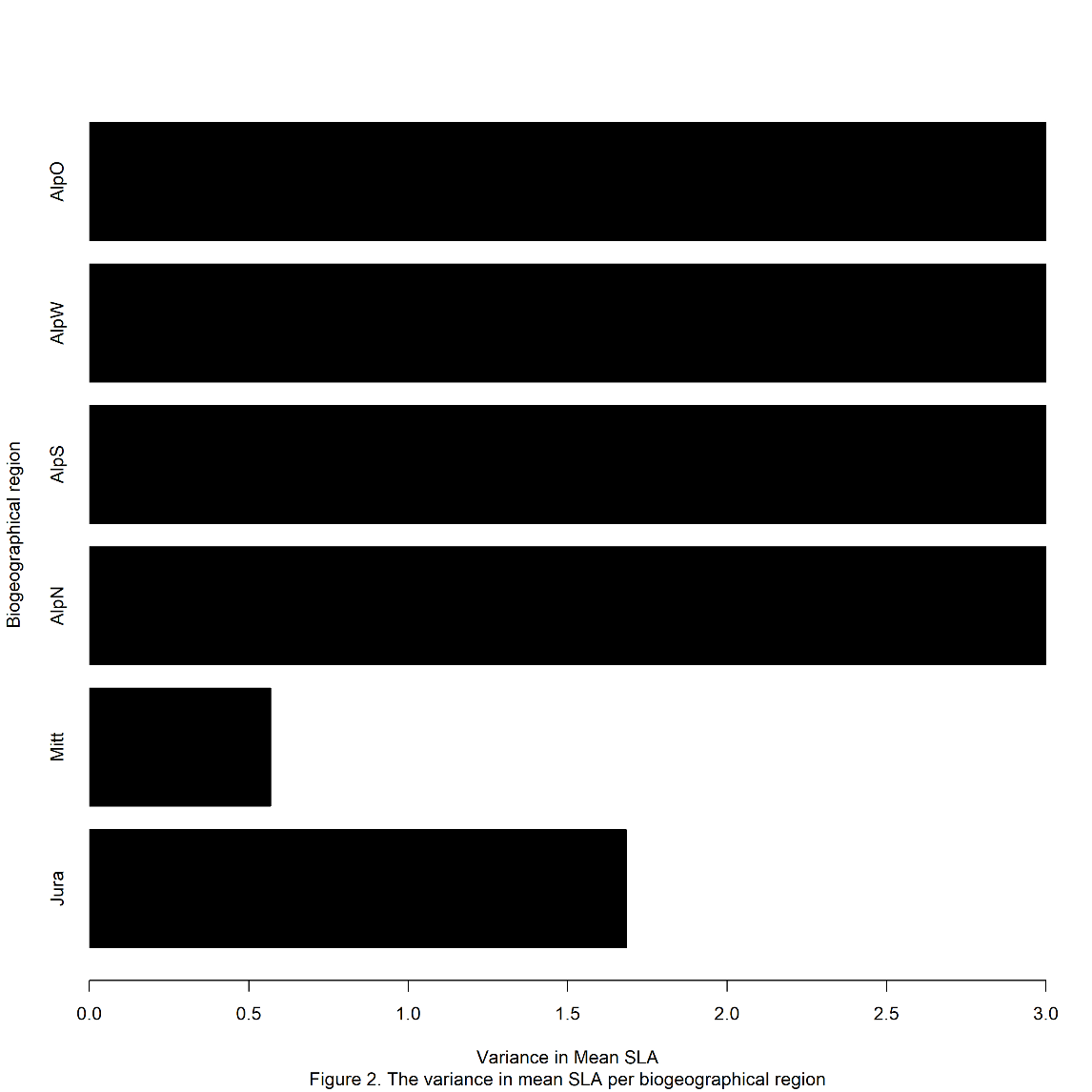

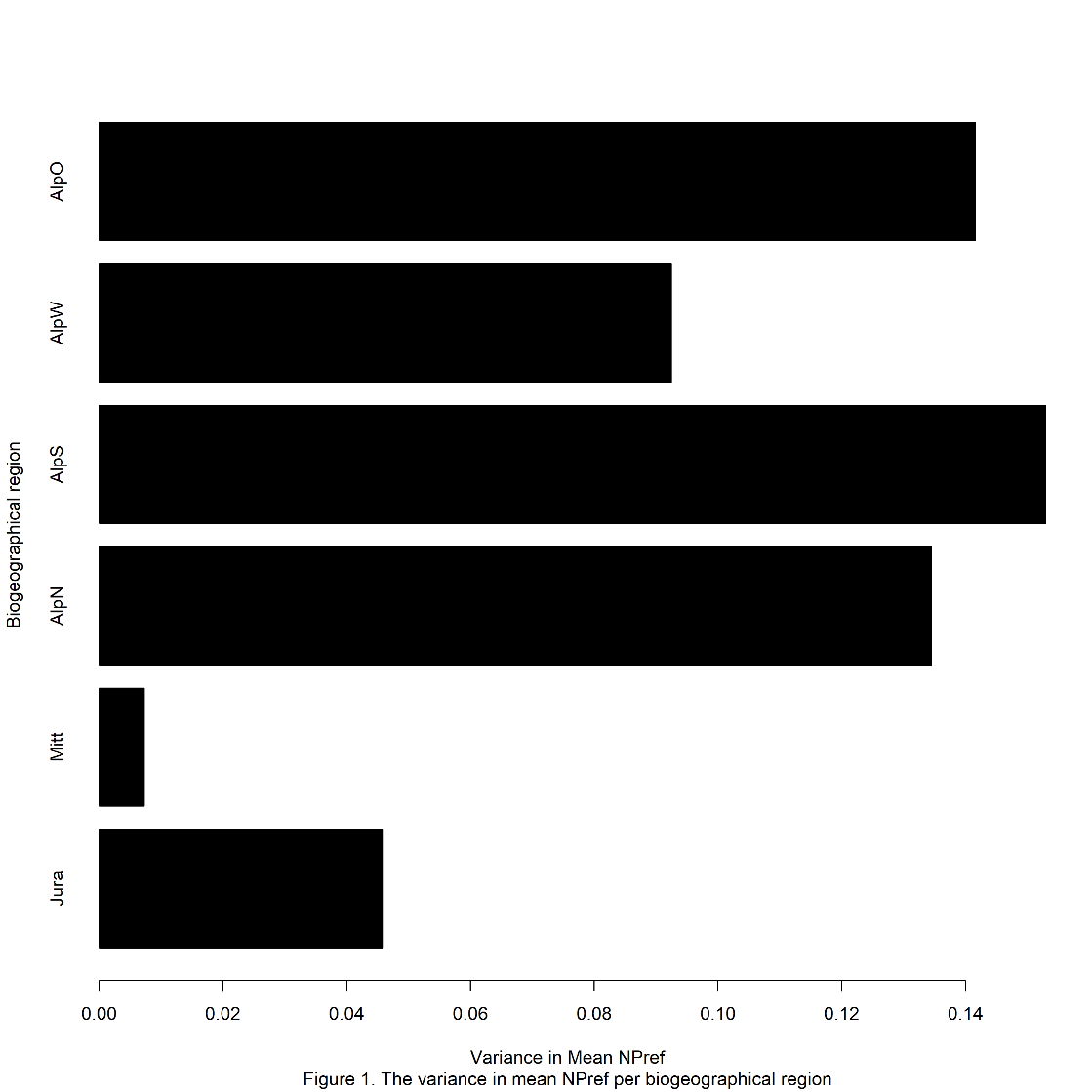

Supplement: Supplementary file 19 — Appendix S1‐S6 [file ECE3-10-9906-s019.docx]
